# Advances in hereditary angioedema in the modern treatment era in China: a focus on diagnosis, treatment, and prognosis

**DOI:** 10.1186/s13023-026-04314-5

**Published:** 2026-03-20

**Authors:** Ye Zhao, Duowu Zou

**Affiliations:** Department of Gastroenterology, Ruijin Hospital, Shanghai Jiao Tong University School of Medicine, Shanghai, China

**Keywords:** Hereditary angioedema, C1-inhibitor, Type 1 HAE, Lanadelumab, Icatibant, Long-term prophylaxis

## Abstract

**Background:**

Hereditary angioedema (HAE) is a rare, potentially life-threatening genetic disorder that is caused by C1-inhibitor (C1INH) deficiency or dysfunction. This scoping review sought to map recent advances in the management of HAE among patients in China.

**Materials and methods:**

A comprehensive literature search was conducted using relevant keywords across the databases PubMed, Embase, the Cochrane Library, ClinicalTrial.gov, and Chinese databases such as the China National Knowledge Infrastructure and Wanfang. The search covered publications from database inception through September 2024. Data was extracted on patient characteristics, disease manifestations, diagnostic approaches, treatments, clinical outcomes, and quality of life (QoL). The selection of articles followed predefined inclusion criteria and was conducted in adherence to the Preferred Reporting Items for Systematic Reviews and Meta-Analyses Extension for Scoping Reviews (PRISMA-ScR) guidelines.

**Results:**

A total of 92 articles were included in this scoping review. Across the literature, the diagnosis of HAE was primarily based on serum complement assessment, particularly measurements of C4 and C1INH levels, along with family history and clinical manifestations. Danazol was reported as the primary treatment in previous studies; however, more recent studies emphasized the increasing use of lanadelumab and icatibant. Icatibant was shown to provide rapid symptom relief during acute HAE attacks, whereas lanadelumab demonstrated effectiveness as a long-term prophylactic therapy by reducing the frequency of attacks. The reviewed studies indicated fewer HAE-related deaths reported in studies published after 2021 compared with previous studies (129 deaths reported up to 2021; five deaths reported between 2021 and September 2024). This reduction may be attributed to increased disease awareness, earlier diagnosis, and advances in therapeutic management. In parallel, several studies reported improvements in angioedema-related quality of life (QoL) scores among patients with HAE in China.

**Conclusion:**

Emerging therapies such as lanadelumab and icatibant are effective in reducing the frequency of attacks and providing rapid symptom relief in Chinese patients with HAE. Nevertheless, further research is warranted to optimize HAE management strategies and to address existing gaps in the evidence within the Chinese population.

**Supplementary Information:**

The online version contains supplementary material available at 10.1186/s13023-026-04314-5.

## Introduction

Hereditary angioedema (HAE) is a rare autosomal dominant disorder characterized by recurrent episodes of cutaneous or submucosal edema, most commonly affecting the skin and gastrointestinal (GI) tract [1]. Attacks affecting the upper respiratory tract are generally less common [1], although 50% of patients experience such an attack at least once in their lifetime [2]. Multiple genetic subtypes of HAE have been recognized [1]. HAE-C1INH-Type 1 and HAE-C1INH-Type 2 are caused by mutations in the *SERPING1* gene, contributing either to reduced C1 esterase inhibitor (C1INH) levels (HAE-C1INH-Type 1) or to normal levels of dysfunctional C1INH (HAE-C1INH-Type 2) [3]. In contrast, HAE with normal C1INH (HAE-nC1INH) is characterized by normal C1INH levels and is associated with mutations in several other genes [4].

HAE is associated with unpredictable attacks and carries the risk of life-threatening laryngeal angioedema, which can significantly impose a substantial physical and psychological burden on patients and their caregivers [1]. Limited disease awareness and restricted access to diagnostic facilities have contributed to underdiagnosis and delayed diagnosis in patients with HAE in China [5]. Failure to diagnose conditions such as intestinal swelling can lead to misdiagnosis, resulting in unnecessary surgical interventions [1]. Moreover, untreated laryngeal attacks can be fatal, with reported mortality rates of up to 40% [2, 6].

In recent years, substantial progress has been made in HAE management in China. The development of HAE guidelines and expert consensus statements has increased disease awareness and contributed to the standardization of patient care [7]. Treatment patterns have also evolved significantly. Before 2021, no HAE-specific therapies for acute attacks were approved in China. For long-term prophylaxis (LTP), attenuated androgens were the most commonly used therapeutic option [6]. The subsequent approval of modern therapies, such as lanadelumab for LTP in 2020 and icatibant for on-demand treatment in 2021, has substantially expanded the range of effective treatment options in China.

Regardless of these recent advancements in the treatment landscape of HAE in China, a comprehensive overview of HAE diagnosis, management, and prognosis in the Chinese population remains lacking. Therefore, this scoping review aims to systematically map and analyze the existing available evidence to understand the current status of HAE in China, emphasizing on diagnostic approaches, treatment strategies, prognosis, and the impact of HAE on QoL. In addition, this review aims to highlight recent paradigm shifts in clinical practice, describe emerging therapeutic strategies of HAE in China, and identify the key evidence gaps to inform future research directions aimed at improving patient outcomes.

## Methods

The scoping review followed a methodological framework including the following steps: defining the research questions and objectives; identifying relevant studies; selecting studies; mapping the data; populating the data, summarizing the data, and reporting the results. This scoping review was prepared in accordance with the Preferred Reporting Items for Systematic Reviews and Meta-Analyses Extension for Scoping Reviews (PRISMA-ScR) guidelines [8].

### Eligibility criteria

All peer-reviewed full-text articles available in English and Chinese were considered eligible for inclusion in this scoping review. Studies were included in the review if they met all of the following inclusion criteria: RCTs/non-RCTs/real-world studies, case studies, or large case series reporting information on the diagnosis, prognosis, treatment, or QoL of patients with HAE, and studies enrolled patients in China (aged ≥ 2 years) diagnosed with HAE and evaluated any therapeutic intervention used for the management of HAE. Studies were included if they reported relevant subgroup analyses, including diagnosis, prognosis, treatment patterns, or QoL outcomes. The articles were excluded if they were reviews, consensuses, theses, questionnaires, and correspondences, cost-related studies, preclinical studies, or studies published in languages other than English and Chinese.

### Literature search

An extensive primary literature search was conducted in PubMed, Embase, and the Cochrane Library, as well as Clinical Trial.gov and major Chinese databases, including the China National Knowledge Infrastructure and Wanfang. The search covered publications from database inception through September 2024 and followed a predetermined search strategy developed previously. We developed a search strategy based on these keywords: “Chinese,” “China,” “Chinese patient,” “hereditary angioedema,” “prevalence,” “diagnosis,” “treatment,” “prognosis,” “biomarkers,” and “quality of life.” Detailed search string is provided in the supplementary file (Appendix 1).

### Study selection

**Fig. 1:**
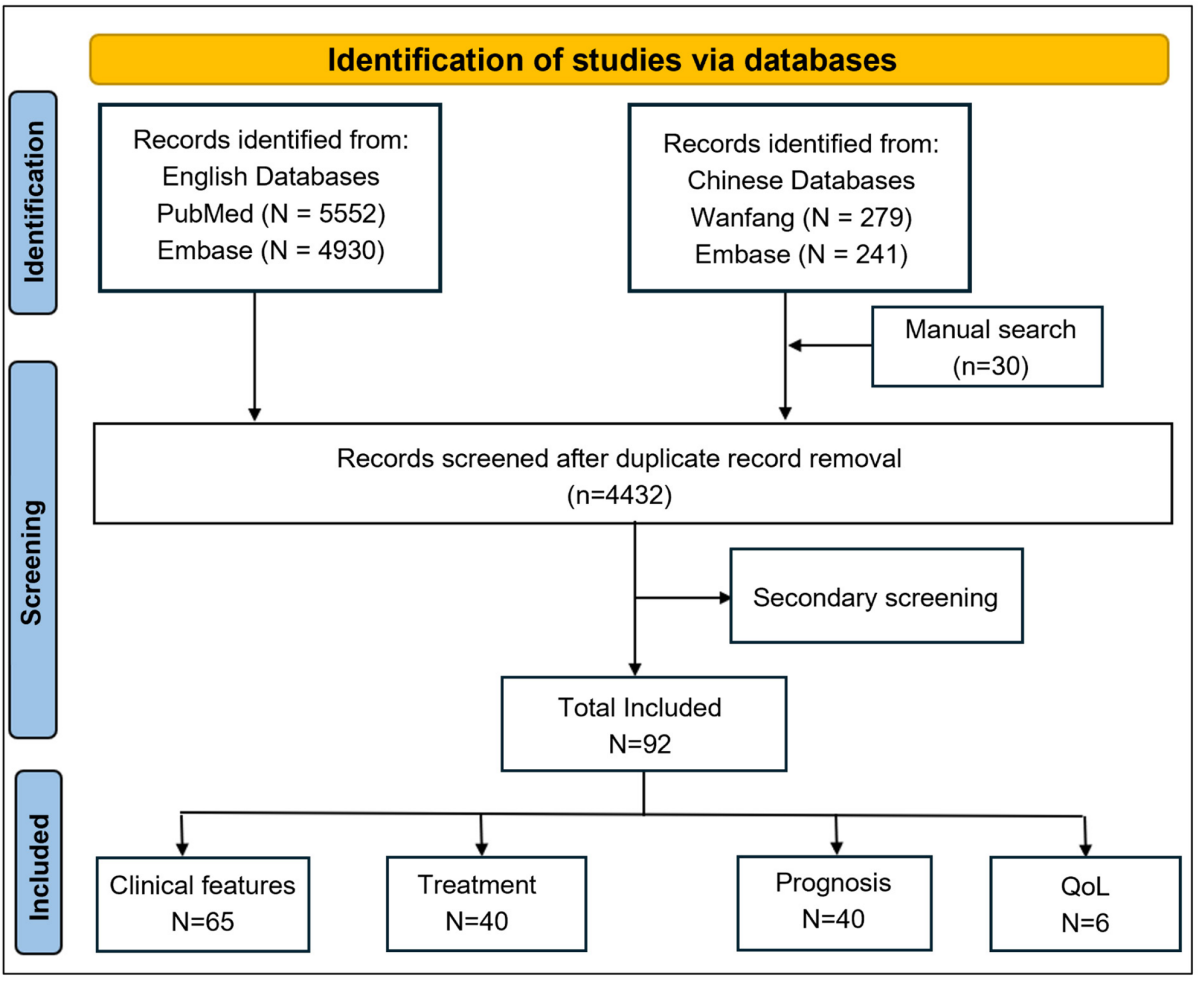
Flow diagram depicting the flow of information through different phases of a scoping review

After the removal of duplicate records, the search results from the databases underwent secondary screening for relevance, and the results including titles without abstract were excluded manually. Two reviewers independently reviewed the titles and abstracts of publications retrieved by the search engine to identify potentially eligible articles based on the predefined exclusion and inclusion criteria. All potentially relevant citations fitting the scope of the present review were requested and inspected in detail using the full-text version. Data extraction was performed using a standardized, predesigned Excel file developed specifically for this review. Any disagreements and requests regarding study eligibility or data extraction were resolved and request for full text review were further adjudicated by involving another reviewer (Fig. 1).

### Data extraction

Data on the following were extracted from eligible articles: title, authors, country of origin, year of publication, sample size, and baseline characteristics, including age, gender, HAE types, family history, comorbidities, attack frequency, diagnostic methods, treatment received, clinical features, prognostic markers, QoL measures, and safety. The Newcastle-Ottawa Scale (NOS) [9] and JADAD quality assessment tools were used to assess the quality of observational and RCT studies [10].

## Results

### Characteristics of the included studies and patients

**Fig. 2:**
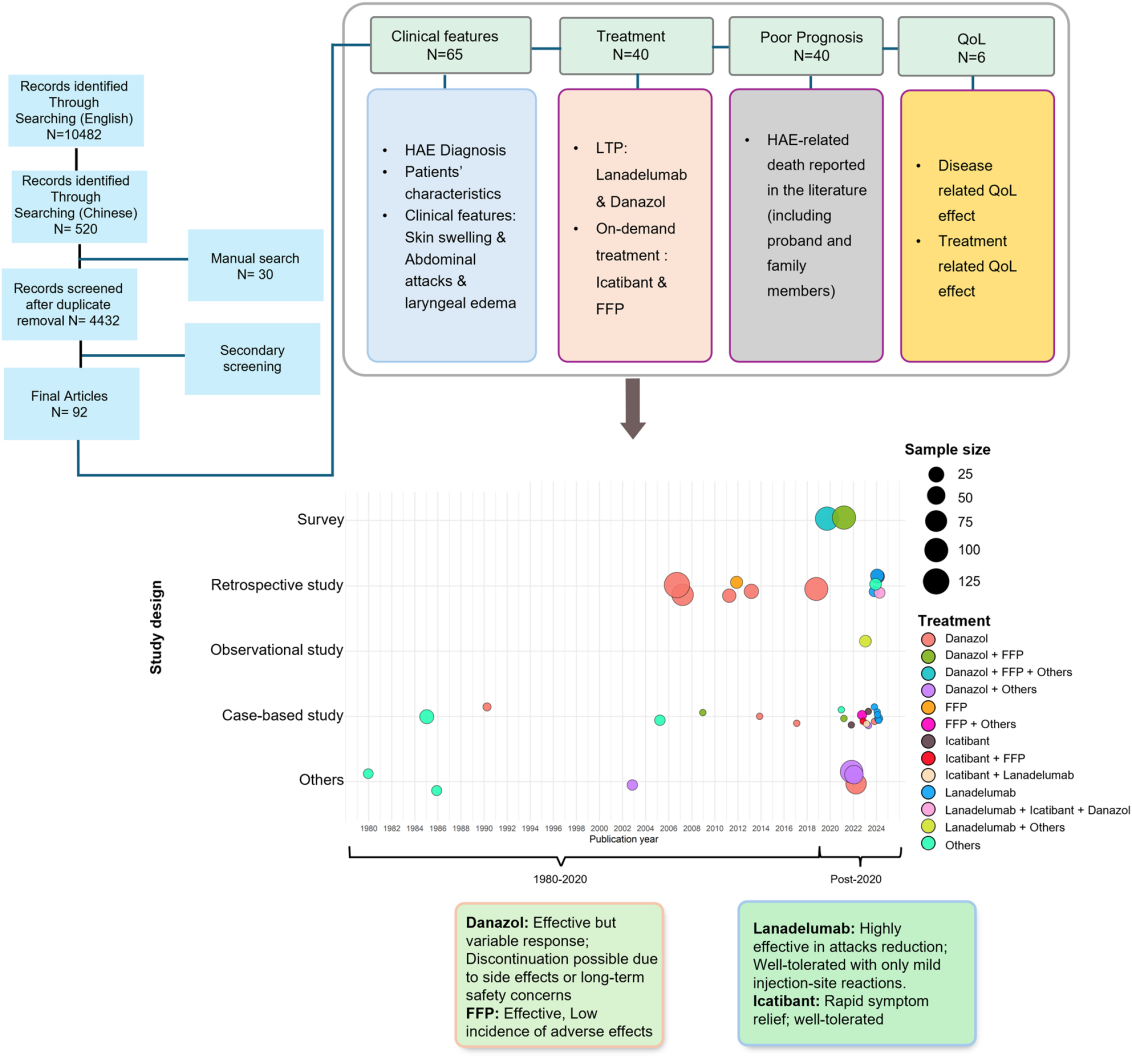
Evidence map summarizing the included studies by publication year (x-axis) and study design (y-axis). Each dot represents an individual study and is positioned according to its year of publication and study design. Dots are placed to distinguish treatments evaluated in the pre-modern therapy era (1980–2020) and the post-modern therapy era (post-2020) among patients with HAE in China. The key aspects assessed are summarized and indicated in the map. When multiple studies share the same year and study design, bubbles may overlap. Overlapping bubbles are displayed in the same color when the evaluated treatment is identical, and in different colors when different treatments are assessed. The size of each bubble reflects the study sample size. The “Others” category includes family studies, genetic analyses, and letters to the editor.Abbreviations: HAE: Hereditary angioedema; QoL: Quality of life; FFP: Fresh Frozen Plasma; LTP: Long-Term Prophylaxis; N: number of patients.

After removing duplicate records, a total of 37 English-language [11–47] and 55 Chinese-language articles [48–102] were included in this scoping review. Among the 92 studies included, the majority were observational studies (42%), followed by case reports (35%) or case series (14%) and other categories (8%), along with a single RCT (1%). The number of patients with HAE ranged from 1 to 158 across different studies, with the possibility of overlapping patients, as many studies originated from the Peking Union Medical College Hospital. Most patients identified in the included studies were diagnosed with type 1 HAE. Across most studies, the diagnosis age of HAE ranged from 6 to 78 years. Baseline characteristics of the study population including gender, age, type of HAE, and family history, are presented in Appendix Table 1. Additionally, a data visualization summarizing the distribution and key characteristics of the included studies is presented in Fig. 2.

### Prevalence

Although the exact prevalence of HAE in China remains unclear, some studies have reported its prevalence. In one of the studies conducted in Taiwan, the estimated prevalence of HAE was approximately 1:550,000 to 1,000,000 individuals [38]. In another report, a low prevalence of HAE in Taiwan was reported (Appendix Table 9) [13] Overall, the available data indicates that HAE is rare among the Chinese population, but population-wide estimates across the studies remain uncertain.

### Diagnosis of HAE in China

#### C1INH *and C4 testing*

Across the included studies, the diagnosis of HAE was primarily based on the assessment of the serum complement profile, particularly measurements of C4 and C1INH levels, along with family history and clinical manifestations (Appendix Table 4). Increased emphasis has been placed on C4 testing in patients suspected of HAE because of its high sensitivity and easy availability. C4 testing was used as a screening tool to identify patients with HAE and reported a prevalence of HAE of 2.43 per 10,000 among patients with reduced C4 levels, and 10.1 per 10,000 among patients with decreased C4 levels of unknown etiology [33]. The data indicates that complement profile assessment, along with clinical evaluation, provided the core diagnostic approach for HAE in China.

#### Gene testing and other biomarkers

Gene mutations in patients with HAE were discussed in 21 studies. Mutations in the *SERPING1* gene were the most frequently identified, reported in 18 studies, while mutations involving the *C1INH* and other genes were reported in 7 studies (Appendix Table 6). Across these studies, identification of 35 novel mutations in *SERPING1*, including nonsense, frameshift, and missense mutations was reported [30]. In addition to *SERPING1*, other mutations in *F12* gene and *PLG* gene, *SERPING1 + MYOF* and *SERPING1 + HS3ST6* gene mutations were identified in specific HAE cases [54].

In addition, potential of plasma protein N-glycomes as noninvasive biomarkers for the diagnosis, monitoring, and prediction of disease severity in patients with HAE was also reported [32].

#### Diagnostic delay

Several previous studies have demonstrated significant diagnostic delays for HAE, with wide variability in the duration of delay. Reported delays ranged from 8 to 39 years in individual cases [96]. However, it is noteworthy that some studies have reported an improvement in diagnostic delays in recent years (Appendix Table 2). Across these studies, a significant reduction in diagnostic delay was observed from 19.75 years before 1999 to 8.67 years between 2000 and 2009, and further reduction to 3.79 years from 2010 to 2017 [14]. These findings suggest a gradual reduction in diagnostic delay over time, although substantial delays remain for many patients.

#### Misdiagnosis of HAE

Misdiagnosis of HAE was frequently reported across the included studies, most often as gastrointestinal (GI) diseases such as gastroenteritis and appendicitis (Appendix Table 5). Patients were also misdiagnosed with conditions such as allergies, urticaria, and other diseases [14]. Misdiagnosis as GI conditions led to unnecessary surgeries in 24.7% of patients [15].

### Clinical features

The most commonly reported clinical manifestations of HAE typically included skin swelling and recurrent abdominal pain. Abdominal symptoms were widely documented across several studies, including abdominal pain, nausea, vomiting, GI tract edema [30, 35], melena [53], ascites [35, 38, 53, 92]. Two studies observed edema of the intestinal wall and extensive thickening of the intestinal wall located in the middle and lower abdomen, with effusion in the abdominal and pelvic areas, detected on abdominal CT scans [90, 93]. Swelling of the duodenum detected on abdominal CT scan was also reported in some cases [11].

Asphyxiation attacks, although less common than other HAE symptoms, have been reported across multiple studies, and were associated with severe consequences. Clusters of both non-fatal and fatal cases have emerged, with some events linked to triggers such as dental procedures [13, 21, 35, 38, 76]. Collectively, these findings highlight the life-threatening risk of asphyxiation in the presence of laryngeal edema and underscore the need for early recognition and prompt intervention.

### Triggering factors

Several triggering factors for HAE were reported across the studies. Frequently reported factors included trauma, compression, stress, emotion, infection, and pregnancy or menstruation. Other triggering factors such as, alcohol intake [27], minor trauma or pressure from activities such as exercise [36], prolonged walking, stressful events, menstrual cycle [37], and tooth extraction [38] were also reported across the studies. Furthermore, emotional changes [13], fatigue [88], and upper respiratory infections [88] were also reported as potential triggers, besides seasonal changes and exposure to cold.

### Treatment approaches

Of the total 92 studies, 40 studies reported treatment approaches for HAE (Appendix Table 7). Before 2021, patients were typically treated symptomatically or with danazol for LTP. The approval of modern treatments has expanded available therapeutic options for patients with HAE. Among the studies included, no eligible studies were observed that particularly evaluated short-term prophylaxis in this patient population.

#### Long-term prophylaxis

##### Lanadelumab

Lanadelumab is recommended as the first-line treatment option for LTP in managing HAE [1]. Ten studies reported the use of lanadelumab for the treatment of HAE attacks. In one study, lanadelumab consistently reduced attack frequency, with mean annual attacks decreasing from 15.2 ± 6.8 (min = 4; max = 25) to 0.3 ± 0.5 (min = 0; max = 1) per year [41]. In this study, some patients (66.7% [4 of 6]) became completely free of attacks after initiating lanadelumab. Reductions of 97.8% in overall attack rate and 98.5% in treated attacks was also observed [41].

Across all studies that used lanadelumab, no serious adverse effects were reported [41, 94]. Injection site pain and injection site erythema were the most common adverse events with lanadelumab [41].

##### Danazol

Several studies (Appendix table Table 7) have reported the use of danazol and its clinical effectiveness in treating HAE. In most cases, the maintenance doses of danazol ranged from 200 to 400 mg/d [13, 20, 38, 53, 65, 76, 79, 92]. Regardless of its effectiveness, danazol treatment was associated with several side effects. Common adverse reactions included increased sebum secretion [20, 29, 76, 77, 79], acne [29, 76, 77], weight gain [15, 20, 29, 76, 77, 79], occasional abdominal pain [79], severe headache [20, 77], and menstrual disorders in female patients [15, 29, 76, 77, 79]. Hepatic related abnormalities such as increase in transaminase [76], detection of liver cyst on B-ultrasound examination were reported in small number of cases [76]. Discontinuation of danazol was reported in some patients, primarily due to the side effects (*n* = 7; 46.7%), pregnancy (*n* = 6; 40%), concerns regarding long-term adverse effects (*n* = 4; 26.7%), unsatisfactory efficacy (*n* = 2; 13.3%), and difficulty adhering to long-term treatment and long-term treatment cost (*n* = 1; 6.7%) [20]. Furthermore, danazol was temporarily replaced with alternative treatment options due to intolerable side effects, [13] whereas the symptoms of HAE were under control in another patient treated with danazol.

#### On-demand treatments

##### Icatibant

Six studies [24, 43, 88, 90, 92, 96] reported the use of icatibant for the treatment of acute HAE attacks. Across these studies, symptom resolution typically occurred within 2 to 14 h. Individual case reports described faster responses in some patients, including symptomatic improvement within 20 min following self-administration [24]. In addition, symptom relief was observed across different types of HAE attacks such as abdomen, skin, and throat, with complete resolution occurring within 4, 16, and 6 h, respectively [96]. Reported side effects were generally mild and transient, such as injection-site pain, erythema, transient dizziness, and forehead sweating [88]. Overall, these findings highlight the effectiveness and safety of icatibant as an acute therapy for HAE.

##### Fresh frozen plasma (FFP)

Seven studies reported improvement or resolution of HAE symptoms after FFP infusion [15, 20, 36, 38, 43, 78, 91]. Across these studies, administration of FFP resulting in a marked reduction in time to complete symptom remission, from a mean of 61.7 (SD ± 27.0) hours before FFP administration to 2.0 (SD ± 12.0) hours after FFP infusion [78]. Adverse reactions to FFP were reported, however, allergic reactions such as fever, chills, and rash occurred in one patient resulting into discontinuation of FFP.

### Mortality associated with HAE

A total of 40 studies were included in the analysis of HAE-related death, collectively reporting 134 deaths. (Appendix Table 3) Of these studies, 35 were published before 2021, whereas only five [33, 42, 52, 89, 91] were published after 2021. Notably, 129 HAE-related deaths were reported in studies before 2021, whereas five deaths were reported across studies after 2021. Two of the included studies were large-scale cohorts, each involving > 100 patients with HAE. Across these cohorts, pharyngolaryngeal edema was reported in 93 patients (58.86%), and 18 patients (11.39%) died from asphyxia caused by laryngeal edema [35] whereas 14 patients died of laryngeal edema in another study [64]. The median age at death from laryngeal edema was 46 years (IQR, 35–53), indicating that HAE may significantly affect the lifespan of patients [27]. Among all included studies, the youngest reported death occurred at age 12 years due to sudden respiratory distress and suffocation [80].

### Impact on QoL

A total of six studies assessed QoL among Chinese patients with HAE (Appendix Table 8) [17–19, 28, 41, 95]. Validated Chinese versions of the Angioedema Activity Score (AAS), Angioedema Quality of Life (AE-QoL) questionnaire, and Angioedema Control Test (AECT) have been demonstrated to be reliable for use in Chinese populations [17]. Chinese patients with HAE consistently demonstrated lower health-related Quality of life (HRQoL) scores compared with the general population. In particular, uncontrolled disease activity and the presence of laryngeal edema were associated with reduced physical and mental well-being [28].

Long-term prophylaxis (LTP) therapies, including lanadelumab and garadacimab, were further associated with improvements in QoL scores and reduced attack frequency in this patient group [41]. After 4 weeks of lanadelumab therapy, Angioedema Quality of Life Questionnaire (AE-QoL) scores including fears/shame domain were significantly reduced, with effects sustained throughout treatment. Better improvement in Dermatology Life Quality Index (DLQI) scores was also observed after 2 weeks [18].

## Discussion

This scoping review synthesizes the evolving evidence on HAE in China, focusing on epidemiology, diagnosis, clinical characteristics, treatment patterns, QoL, and prognosis. In recent decades, substantial progress in HAE management observed in China, largely driven by improved disease awareness, earlier diagnosis, and the introduction of targeted therapies. These advances have led to measurable improvements in patient outcomes and HAE management in China.

In the Asia-Pacific region, a minimal prevalence of HAE of 0.02 per 100,000 population has been reported, with substantial heterogeneity across centers [103]. However, the estimated prevalence of approximately 1.6 per 100,000 population reported in Western countries [103]. This difference is likely multifactorial, reflecting underdiagnosis, delayed diagnosis, and possible interpopulation and interethnic variation in disease prevalence [33, 103]. This scoping review highlights a gap of HAE data in China being largely available within specialized centers, limiting the scope of epidemiologic data and underscoring the need for population‑based surveillance strategies.

The first documented case of HAE in China was reported in 1980 [100]. Despite this early recognition, owing to the rarity of the disease, HAE remains under-recognized in many clinical settings, and the published evidence base is largely limited to isolated case reports. Diagnostic testing and systematic research on HAE in China have been primarily conducted at the Peking Union Medical College Hospital [16, 20, 21, 27], due to limited diagnostic facilities available, significant underdiagnosis or delay in the diagnosis in HAE in China over recent decades [104]. Across the included studies, diagnostic delay remains a prominent challenge in the Chinese population with HAE with a median diagnostic delay of 11.04 years (IQR, 6.06–18.27) [14]. The median diagnostic delays reported in a multicenter European study involving data from eight countries was 8.5 years (IQR, 0–62) [105]. The lack of centralized laboratory testing capabilities for C1INH function, along with the limited number of laboratories and hospitals nationwide capable of conducting C1INH level testing, hinders timely diagnosis [33, 106]. In earlier findings it was indicated that delayed diagnosis is not only due to the rare nature of the disease, but also due to the overlapping of HAE symptoms such as common allergic, gastrointestinal, and systemic conditions [106]. Additionally, evidences from earlier studies reported a progressive reduction in diagnostic delay over time, may be attributed to increased clinician awareness, increasing screening initiatives, and improved access to laboratory diagnostics [2, 14].

The mapped evidence indicates that in China, serum C4 testing is frequently used as an initial screening tool for HAE. Its value lies not only in its relatively high sensitivity [107, 108] but also in its broad availability across hospitals by contrast, access to confirmatory C1INH testing (including both antigenic protein levels and functional assays) remains limited in many institutions, meaning that C4 often serves as the most readily accessible first-step test in routine practice. However, it is crucial to note that normal serum C4 levels do not exclude HAE. When clinical suspicion is high, both C1INH antigenic and functional levels should be measured to definitively confirm or exclude HAE-C1INH [109, 110]. In addition, D-dimer levels during acute HAE attacks may provide supportive diagnostic evidence, as these levels can increase during attacks. The growing interest in genetic testing and exploratory biomarkers reflects a shift toward precision diagnostics, but their current use remains largely investigational and geographically constrained. Taken together, the diagnostic literature suggests that availability, rather than diagnostic optimality, continues to shape real-world practice.

Despite methodological heterogeneity, the clinical features of HAE reported in Chinese patients are largely consistent with global findings, with the most common symptoms being swelling of the face, extremities, and skin. However, abdominal attacks appear to be less frequently reported in Chinese cohorts, which may reflect the under-recognition or delayed reporting observed across studies [2]. Several triggering factors were identified in Chinese population, including trauma, physical activities, stress, and menstrual cycle. Earlier studies, among Hungarian populations have reported similar triggering factors such as exertion stress and trauma indicating that these patterns may be applicable across populations [111]. Owing to the multifactorial symptoms of HAE, differentiating this condition from other diseases with overlapping symptoms remains challenging. Therefore, it is important for healthcare practitioners to maintain a high level of awareness of HAE and to distinguish it from other diseases such as histaminergic angioedema to ensure appropriate treatment and optimize patient outcomes [112].

In China, officially approved therapeutic options include LTPs such as lanadelumab, danazol, and tranexamic acid, as well as on-demand treatments such as icatibant and FFP [2, 20, 41]. The present scoping review indicated that danazol was the most frequently used treatment in China prior to 2020. However, its side effects, such as weight gain, menstrual disorders, and liver dysfunction, remain significant challenging that led to treatment discontinuation or switching. In consistent with the previous studies from European population have indicated the global challenge of optimizing the efficacy of these therapies while reducing the associated adverse effects [113].

The approval of modern targeted therapies, such as lanadelumab and icatibant, as first-line treatments for LTP and on-demand use, respectively, in China has shown promising outcomes for patients with HAE, and signals a transition toward pathophysiology-driven treatment strategies [41]. Although the current evidence base is limited in size, the consistency of reported benefits across attack frequency, symptom resolution, and functional outcomes aligns with global evidence [114–116]. Moreover, effective attack prevention and symptom management can substantially enhance the QoL of patients with HAE. Similarly, improvements in QoL reported in recent studies highlight how effective HAE management leads to psychological well-being, social functioning, and disease control [18, 19]. Furthermore, C1INH replacement therapy has been used globally for both acute and prophylactic treatment management of HAE. However, at present, it has not been officially approved in mainland China. There are ongoing clinical trials on plasma-derived and recombinant human C1INH, and it is anticipated that more therapies will become available in the future.

Recent studies in China indicate a downward trend in HAE-related mortality, which may be a result of the beneficial effect of early diagnosis and newer treatments. Similar trends have been reported worldwide, with more effective management resulting in improved outcomes. Nonetheless, mortality data in this review should be interpreted as descriptive and hypothesis generating, given the limited number of studies and reported events [18, 117, 118].

Although this scoping review provides a comprehensive overview of HAE management in China, a few limitations should be acknowledged. First, a significant number of the included studies on HAE in China originated from a limited number of centers, particularly Peking Union Medical College Hospital. Therefore, there is a possibility of patients overlap across studies. In the absence of detailed reporting on recruitment periods and individual patient characteristics, it was challenging to exclude duplicates from the record, which may offer duplication bias and influence the final results. Future studies should clearly report recruitment timelines and population characteristics to avoid potential overlapping of data. In addition, there are limited large-scale data on patients with HAE in China, as much of the available evidence is derived from case reports or small observational studies. Furthermore, although the risk of bias among the included studies was assessed wherever applicable, a comprehensive assessment for all included studies such as web-based surveys, case reports, and case series was not possible because of the lack of validated assessment tools applicable across these study designs. Therefore, the overall strength of the evidence is limited by heterogeneity present in study designs and methodology, including small population size and the lack of control groups in many studies, which can affect internal validity. However, as the purpose of this scoping review was to map the existing evidence, characterize the research landscape of HAE in China, and identify knowledge gaps, these limitations are inherent to the nature of the available literature, highlighting the need for future research and systematic studies to strengthen the evidence and inform clinical practice.

## Conclusions

Patients with HAE in China continue to face considerable challenges related to delayed diagnosis and limited access to optimal treatment. This scoping review highlights significant progress in the management of HAE in China, addressing aspects of prevalence, diagnosis, treatment, and QoL outcomes. Although HAE remains a rare and often misdiagnosed condition in China, the therapeutic landscape, including lanadelumab for LTP and icatibant for on-demand treatment, has shown promising outcomes. Regardless of these advances, future studies are warranted to explore the specific characteristics of HAE in Chinese patients and develop targeted strategies to enhance the diagnosis and management of this rare disease.

## Supplementary Information

Below is the link to the electronic supplementary material.

Supplementary Material 1

Supplementary Material 2

## Data Availability

The original contributions presented in the study are included in the article; further inquiries can be directed to the corresponding author.

## Abbreviations

AE-QoL: Angioedema quality of life
C1INH: C1 esterase inhibitor
C4: Complement 4
CT: Computed tomography
DLQI: Dermatology Life Quality Index
F12: Factor XII
FFP: Fresh frozen plasma
GI: Gastrointestinal
HAE: Hereditary angioedema
HS3ST6: Heparan sulfate-glucosamine 3-O-sulfotransferase 6 genes
STP: Short-term prophylaxis
LTP: Long-term prophylaxis
IQR: Interquartile range
QoL: Quality of life
MYOF: Myoferlin
HAE-nC1INH: HAE with normal C1INH
PRISMA-ScR: Preferred Reporting Items for Systematic Reviews and Meta-Analyses Extension for Scoping Reviews
SD: Standard deviation
SERPING1: Serpin family G member 1
