# Supplementary material for: Advances in hereditary angioedema in the modern treatment era in China: a focus on diagnosis, treatment, and prognosis
Source: Orphanet J Rare Dis. 2026 Mar 20;21:171. doi: 10.1186/s13023-026-04314-5 (PMC13127019; doi:10.1186/s13023-026-04314-5)
Supplement: Supplementary file 1 — Supplementary Material 1 [file 13023_2026_4314_MOESM1_ESM.docx]

**Appendix 1.**

**Search strings:**

**Prognosis**

((("Hereditary angioedema") OR (HAE)) AND ((((((((((prognosis) OR (Biomarkers)) OR ("plasma protein N‐glycome")) OR ("C1-INH")) OR ("C1 complex")) OR ("C1-INH complex")) OR ("Mannan-binding lectin-associated serine protease-1")) OR ("C4 levels")) OR ("Epidermal Growth Factor")) OR (("C1-INH") AND ("IgM antibodies")))) AND (China)

**Treatment**

((("Hereditary angioedema") OR (HAE)) AND (((((((((("C1 esterase inhibitor concentrate") AND ("plasma-derived")) OR ("icatibant acetate")) OR ("ecallantide")) OR ("recombinant human C1 esterase inhibitor")) OR ("lanadelumab")) OR ("danazol")) OR ("tranexamic acid")) OR ("Fresh frozen plasma")) OR ("garadacimab"))) AND (China)

**Diagnosis**

((((((((((((Diagnosis) OR ("Genetic testing")) OR ("C4 level")) OR ("C1-INH level")) OR ("C1q level")) OR ("Factor XII assay")) OR ("C1-INH gene mutation")) OR ("F12 gene mutation")) OR ("Bradykinin test")) OR ("C1 esterase inhibitor")) OR ("IgE levels")) AND (("Hereditary angioedema") OR (HAE))) AND (China)

**QOL**

(("Hereditary angioedema") OR (HAE)) AND (((((((("quality of life") OR ("Health-Related Quality of Life")) OR ("Life Quality")) OR ("activities of daily living")) OR ("patient satisfaction")) OR ("caregiver burden")) OR ("quality adjusted life year")) OR ("Angioedema Quality of Life Questionnaire"))

**Clinical features**

(("Hereditary angioedema") OR ("HAE")) AND (((((((("clinical feature") OR ("clinical presentation")) OR ("clinical manifestation")) OR ("skin swelling")) OR ("gastrointestinal (GI) symptoms")) OR ("pharyngeal swelling")) OR ("laryngeal swelling")) OR ("genital swelling")) OR ("swelling")) AND (China)

**Prevalence**

((("Hereditary angioedema") OR (HAE)) AND (Prevalence)) AND (China)
