# Supplementary material for: Advances in hereditary angioedema in the modern treatment era in China: a focus on diagnosis, treatment, and prognosis
Source: Orphanet J Rare Dis. 2026 Mar 20;21:171. doi: 10.1186/s13023-026-04314-5 (PMC13127019; doi:10.1186/s13023-026-04314-5)
Supplement: Supplementary file 2 — Supplementary Material 2 [file 13023_2026_4314_MOESM2_ESM.docx]

| **Appendices**  **Tables:**  **Appendix Table 1 Summary of patients’ characteristics based on 92 included studies** | | | | | | |
| --- | --- | --- | --- | --- | --- | --- |
| **Author name, year** | **Study design** | **Total study population (n)** | **Gender** | **Age (years)** | **HAE type (n)** | **Family history** |
| Xie, 1980 [50] | Family study | HAE = 3 in 1 family | M = 3 | Case 1: 31  Case 2: 21 | NA | Yes |
| Zhou, 1980 [100] | Family study | HAE = 5 in 1 family | M = 1  F = 4 | Case 1: 53 | NA | Yes |
| Huang, 1983 [101] | Family study | HAE = 4 in 1 family | M = 1  F = 3 | Case 1: 28 | NA | Yes |
| Zhang, 1985 [102] | Family study | HAE = 25 in 6 families  Alive HAE = 22 | M = 12  F = 10 | R: 4–74  Average: 33 | NA | Yes |
| Qian, 1986 [99] | Family study | HAE = 6 in 3 families | M = 3  F = 3 | R: 24–53 | NA | Yes |
| Qian, 1988 [63] | Family study | HAE = 6  Healthy control = 40 | M = 3  F = 3 | R: 26–55  Average: 39 | NA | Yes |
| Zhang, 1988 [98] | Family study | HAE = 14 in 1 family | M = 5  F= 9 | Case 1: 41  Case 2: 62 | NA | Yes |
| Lin, 1989 [62] | Case report | 1 | F = 1 | 59 | NA | Yes |
| Zhang, 1990 [64] | RCT | HAE = 26 | M = 13  F = 13 | R:22–63 | NA | NA |
| Qian, 1990 [65] | Case report | 2 | M = 2 | Case 1:56  Case 2: 50 | NA | Yes |
| Pu, 1991[66] | Family study | HAE = 2 in 2 families | M = 1  F = 1 | Case 1: 29 Case 2: 27 | NA | Yes |
| Lu, 1994 [67] | Observational study | 12 | M = 6  F = 6 | R:17–65  Average: 39.6 | Type 1 = 12 | Yes (n = 10) |
| Xie, 1994 [68] | Family study | HAE = 3 in 1 family | M = 3 | Case 1 = 69  Case 2 = 31  Case 3=21 | NA | Yes |
| Wang, 1995 [69] | Family study | HAE = 53 in 12 families | M = 24  F = 26 | 34.3 (4–74) | NA | Yes |
| Wang, 1995 [70] | Family study | HAE = 5 in 1 family | M = 2  F= 3 | Case 1: 34 | NA | Yes |
| Zhou, 1996 [71] | Family study | HAE = 10 in 1 family | M = 5  F = 5 | Case 1: 43 | NA | Yes |
| Choy, 1997 [22] | Case Report | 2 | M = 1 F = 1 | Case 1: 30  Case 2: 65 | Case 1: NA Case 2: Type 1 | Yes (n = 2) |
| Lu, 2001 [72] | Case report | 1 | M = 1 | 32 | NA | Yes |
| Zhi, 2003 [48] | Family study | HAE = 7 in 1 family;  healthy control=30 | M = 4  F = 3 | Proband: 45 | Type 1 = 5 | Yes |
| Huang, 2005 [23] | Case Report | HAE cases: 7  (family members: 11) | M = 5  F = 2 | 33  R: 5–69 (n = 11) | Type 1 | Yes |
| Ashrafian, 2005 [47] | Case Report | 5 | M = 5 | NA | NA | Yes |
| Ren, 2005 [73] | Prospective study | HAE = 23 Control = 37 | NA | NA | NA | NA |
| Sheng, 2005 [74] | Family study | HAE = 5 in 1 family | M = 2 F = 3 | Case 1: 75  Case 2: 74 | NA | Yes |
| Peng, 2006 [75] | Family study | HAE = 6 in 1 family | M = 2  F = 4 | Case 1: 27 | NA | Yes |
| Wen, 2007 [12] | Case study | 15 (family members) | F (proband) | 16 (proband) | Type 1= 6 | Yes |
| Ren, 2007 [76] | Retrospective study | 133 | M = 69  F = 64 | R: 2–72 | Type 1 = 130  Type 2 = 3 | Yes (n = 125) |
| Zhi, 2007 [77] | Retrospective study | 86 | NA | NA | NA | Yes (n = 79) |
| Ren, 2007 [61] | Observational  study | HAE = 23 Healthy control = 40 | M = 15  F = 8 | 41 ± 18  R: 16-72  (HAE group) | Type 1=23 | NA |
| Tang, 2009 [36] | Case report | 1 | F = 1 | 20 | NA | Yes |
| Lei, 2011 [13] | Retrospective study | 19 | M = 11  F = 8 | R: 5–69  28.16 ± 15.64 | Type 1 = 19 | Yes (n = 19) |
| Xu, 2012 [16] | Genetic survey | 48 | NA | NA | Type 1 and 2 | NA |
| Qu, 2012 [39] | Case report | 11 | F = 7  M = 4 | Proband age: 61 III generation  1^st^ patient: 61 3^rd^ patients: 61  IV generation  3^rd^ patient: 40 7^th^ patients: 30 | Type 1 | NA |
| Tang, 2012 [78] | Retrospective study | 13 | M = 7  F = 6 | 30 ± 17 | Type 1 = 5  Type 2 = 1  Unknown = 7 | NA |
| Xu, 2013 [35] | Retrospective study | 158 | M = 78  F = 80 | NA | Type 1 = 156  Type 2 = 2 | NA |
| Tang, 2013 [79] | Retrospective study | 24 | M = 12  F = 12 | R: 11–70  36±15 | NA | NA |
| Xu, 2013 [81] | Observational study | HAE = 7  Healthy control = 53 | NA | NA | Type 1 = 7 | NA |
| Huang, 2013 [80] | Case report | 1 | F = 1 | 60 | NA | Yes |
| Xu, 2014 [21] | Observational study | HAE (with UAE) = 43  HAE (without UAE) = 29  HC = 50 | M = 22  F = 21  (HAE with UAE) | NA | Type 1 = 42  Type 2 = 1 | Yes (n = 43) |
| Zhang, 2014 [82] | Family study | HAE = 15 in 1 family | M = 5  F = 10 | Proband: 58 | Proband: Type 1 | Yes |
| Hu, 2014 [83] | Case report | 1 | F = 1 | 32 | NA | Yes |
| Wu, 2015 [84] | Case report | 1 | M = 1 | 57 | NA | Yes |
| Liu, 2017 [37] | Case Report | 1 | F = 1 | 26 | Type 1 | Yes |
| Li, 2017 [85] | Family study | HAE = 6 in 1 family | M = 4  F = 2 | Case 1: 52 Case 2: 61 | NA | Yes |
| You, 2017 [86] | Case report | 1 | F = 1 | 21 | NA | Yes |
| Liu, 2019 [14] | Internet-based survey | 96 | M = 43  F = 53 | R: 11–30 y = 29  R: 31–44 y = 46  R: 45–68 y = 21 | Type 1= 92  Type 2= 4 | Yes (n = 64) |
| Liu, 2019 [28] | Observational study | 104 | M = 47  F = 57 | R: 18–30 y = 27  R: 31–44 y = 52  R: ≥ 45 y = 25 | Type 1= 101  Type 2= 3 | Yes (n = 72) |
| Wong, 2019 [40] | Case Report | 1 | F = 1 | 28 | Type 1 | Yes |
| Qu, 2019 [87] | Family study | HAE = 10 in 1 family | M = 6  F = 4 | Proband: 51 | NA | Yes |
| Liu, 2020 [20] | Cross-sectional survey | Total = 107 | M = 49  F = 58 | R: 11–20 y = 4  R: 21–30 y = 26  R: 31–40 y = 40  R: ≥41 y = 37 | Type 1 = 103  Type 2 = 4 | Yes (n = 74) |
| Cao, 2020 [27] | Web-based survey | 103 | M = 47  F = 56 | R: 15–68  Mean = 37.7 | Type 1 | Yes |
| Xu, 2020 [34] | Genetic analysis | HAE = 9 HC = 53 | M = 3  F = 6 | R: 25-81 | Type 1 | NA |
| Cao, 2021 [15] | Web-based survey | 107 | M = 49  F = 58 | R: 11–68 Mean = 38.2 | Type 1= 103  Type 2 = 4 | Yes (n = 53) |
| Zhang, 2021 [32] | Observational study | Discovery cohort: untreated HAE = 21;  treated HAE = 12 HC = 50  Validation cohort: untreated HAE = 18; treated HAE = 30 HC = 40 | Discovery cohort: M=39  F = 44  Validation cohort:  M = 26 F = 62 | 33.1 ± 11.0 41.5 ± 13.4 35.6 ± 7.7  43.3 ± 11.9 42.6 ± 12.9 42.5 ± 9.9 | Discovery cohort:  Untreated type‐1 HAE = 21  Treated type‐1 HAE = 12  Validation cohort:  Untreated type‐1 HAE = 18  Treated type‐1 HAE patients = 30 | NA |
| Lin, 2021 [38] | Case report | 1 | F = 1 | 31 | Type 1 | NA |
| Huang, 2021 [11] | Case report | 1 | F = 1 | 27 | Type 1 | NA |
| Wong, 2022 [19] | Prospective study | Total = 44 HAE-index=15 HAE-screened=29 | M=20  M= 6  M=14 | Median = 47.5 (37.8-56.3) Median = 44.0 (37.0–50.0) Median= 50.0 (39.0–57.0) | NA | NA |
| Xu, 2022 [29] | Retrospective study | 74 | M=35  F= 39 | R: 16–71 | Type 1= 73  Type 2= 1 |  |
| Wang, 2022 [30] | Observational study | 97 | M=41  F=56 | 36.0±12.7 | Type 1= 91  Type 2= 6 | Yes (n=64) |
| Cui, 2022 [33] | Single-center, non-interventional study | Total: 8226 (C4 decreased subjects) Confirmed HAE = 2 | M = 1  F = 1 | M = 51  F = 35 | Type 1 | Yes (patient 2) |
| Wang, 2022 [45] | Observational study | Drug-naïve HAE patients = 34   HC = 82 | M=9  F=25 | 44.2 ± 10.7 | - | NA |
| Cao, 2022 [46] | Observational study | HAE = 55 HC = 27 | M = 20  F = 35 | R: 12–71  Median 40 | Type 1 = 46 Type 2 = 9 | NA |
| Yao, 2022 [88] | Case report | 1 | M = 1 | 47 | NA | Yes |
| Jia, 2022 [89] | Family study | HAE = 9 in 1 family | M=4  F=5 | Patient alive with symptoms (n = 5)  R: 26–78  Proband: 48 | Patient alive  Type 1 = 7  Type 2 = 1 | Yes |
| Wong, 2023 [18] | Prospective study | Total = 11 Garadacimab = 8 Lanadelumab = 3 | M = 5  F = 6 | 42.5 ± 13.4 | Type 1 = 9  Type 2 = 2 | NA |
| Li, 2023 [24] | Letter to the Editor | 1 | F = 1 | 57 | Type 1 | Negative |
| Lu, 2024 [26] | Case report | 1 | F = 1 | 53 | Type 1 | NA |
| Luo, 2023 [43] | Case review | 1 | M = 1 | Early 20s | NA | Negative |
| Wong, 2023 [44] | Case report | 1 | M = 1 | 37 | Type 1 | Yes |
| Peng, 2023 [90] | Case report | 1 | M = 1 | 27 | NA | Yes |
| Zhang, 2023 [91] | Case report | 4 in 1 family | M = 1  F = 3 | 23–55 | NA | Yes |
| Mak, 2024 [17] | Questionnaire-based survey | Total = 118  CSU = 95  HAE = 23 | M = 27  F = 91 | R: 21–89 Median=50 | Type 1 = 20  Type 2 = 3 | NA |
| Lu, 2024 [25] | Case study | 1 | F = 1 | 48 | NA | Yes |
| Jindal, 2024 [31] | Observational study | HAE = 14 Chinese, 17 Indian Matched controls = 31 | M: F = 1:1.6 (Ratio) | R: 5–77  Median = 40 | Type 2 = 31 | NA |
| Yao, 2024 [41] | Retrospective  observation study | 6 | M = 3  F = 3 | 36 ± 11.8 | Type 1 = 5  Type 2 = 1 | Yes (n=5) |
| Liu, 2024 [42] | Perspective | 1 | M=1 | 22 | NA | Yes |
| Wang, 2024 [92] | Retrospective study | 8 | M=1  F =7 | 45.9 ± 13.0 | Type 1= 8 | Yes (n = 7) |
| Wang, 2024 [93] | Case report | 1 | F = 1 | 36 | NA | Yes |
| Gao, 2024 [94] | Retrospective study | 18 | M = 6  F = 12 | NA | Type 1= 14  Type 2 = 1  Type 3 (nC1INH) = 3 | Yes (n = 8) |
| Zhang, 2024 [95] | Retrospective study | 11 | M = 4  F = 7 | R: 21–65  35.27 ± 12.92 | NA | NA |
| Yao, 2024 [96] | Retrospective study | 20 | M = 12  F = 8 | Mean = 43.8 | Type 1/Type 2 = 18  Type 3 (nC1INH) = 2 | NA |
| Li, 2024 [97] | Family study | HAE = 13 in 1 family | NA | NA | Type 1= 13 | Yes |
| Du, 2024 [60] | Case series | 4 | M = 2  F = 2 | 16- 36 | Type 1= 3  Type 2= 1 | NA |
| Zhou, 2024 [53] | Case report | 1 | M = 1 | 40 | NA | NA |
| Xu, 2024 [54] | Retrospective study | 13 | NA | NA | Type 1 = 2  Type 3 (nC1INH) = 1  Unknown = 10 | NA |
| Wei, 2024 [55] | Retrospective study | HAE = 1  Suspected patients = 5 | NA | NA | Type 3 (nC1INH) | Yes |
| Ji, 2024 [56] | Prospective study | HAE = 48 Control = 32 | NA | Mean = 40  (all groups) | NA | NA |
| Cao, 2024 [51] | Prospective study | HAE stable phase = 20 HAE recent attack = 16 HAE acute attack phase = 7 | NA | NA | NA | NA |
| Li, 2024 [57] | Family study | HAE = 10 in 2 families | M = 5  F = 5 | NA | Type 1 = 10 | NA |
| Yu, 2024 [58] | Retrospective study | 8 | M = 3  F = 5 | R: 17-57  Mean=37 | Type 1 = 7  Type 2 = 1 | Yes (n = 8) |
| Jiang, 2024 [59] | Case report | 2 | M = 2 | Case 1=17  Case 2=31 | Type 1 =1 Type 2 =1 | Yes (n = 1) |
| Zhang, 2024 [49] | Case report | 1 | F = 1 | 17 | Type 1 | No |
| Xia, 2024 [52] | Family study | HAE = 10 in 2 families | M = 6  F = 4 | Proband 1 = 12   Proband 2 = 17 | Proband 1 = Type 2  Proband 2 = Type 1 | Yes |

*F,* female*; HAE,* hereditary angioedema; *HC,* healthy control; *M,* male; *NA,* not available; *n*C1INH*,* normal C1 esterase inhibitor*; R,* range

**Appendix Table 2 Disease characteristics reported across studies**

| **Author name, year** | **Groups** | **Disease duration**  **(years)** | **Age at symptom onset**  **(years)** | **Diagnostic delay**  **(years)** | **Attack frequency** | **Severity** **score** |
| --- | --- | --- | --- | --- | --- | --- |
| Xie, 1980 [50] | NA | 1–22 | NA | NA | NA | NA |
| Zhou, 1980 [100] | NA | 20 | 33 | NA | NA | NA |
| Huang, 1983 [101] | NA | 25 | 3 | NA | NA | NA |
| Zhang, 1985 [102] | NA | NA | 1–10 years: 6  11–20 years: 12  21–25 years: 4 | NA | A few days to several years  7 cases reported frequent attacks (> 2 times/month) | NA |
| Qian, 1986 [99] | NA | NA | 14–21 | NA | Hospitalizations ranged from 1 to 37, with an average of 8 | NA |
| Qian, 1988 [63] | NA | NA | 12–14 years: 4  > 20 years: 2 | NA | A few days to several years | NA |
| Zhang, 1988 [98] | NA | Case 1: 23  Case 2: 30 | Case 1:18  Case 2: 32 | NA | NA | NA |
| Lin, 1989 [62] | NA | 20 | 39 | NA | NA | NA |
| Qian, 1990 [65] | NA | Case 1: NA  Case 2: 30 | Case 1: since childhood  Case 2: 20 | NA | Before medication: laryngeal swelling  Case 1: 2–3 times/year  Case 2: once/10 years | NA |
| Pu, 1991 [66] | NA | Case 1: 19  Case 2: 22 | Case 1: 10  Case 2: 5 | NA | Case 1: 6 times in 2 years  Case 2: 3~7 times/year | NA |
| Wang, 1995 [70] | NA | >10 |  | NA | NA | NA |
| Zhou, 1996 [71] | NA | > 30 | 11 | NA | NA | NA |
| Choy, 1997 [22] | NA | NA | Case 1: 3 | NA | NA | NA |
| Lu, 2001 [72] | NA | 14 | 18 | NA | NA | NA |
| Zhi, 2003 [48] | NA | 21 | 24 | NA | Once/month | NA |
| Huang, 2005 [23] | NA | NA | R: 5–30  Case: 27 | NA | NA | NA |
| Ashrafian, 2005 [47] | NA | NA | Case Ca had laryngeal edema at the age of 10 years | NA | NA | NA |
| Peng, 2006 [75] | NA | 22 | NA | NA | Once every 3–5 months | NA |
| Wen, 2007 [12] | NA | NA | 14–16 | NA | Proband: 3 attacks in 2 years; 12-year-old sister had 2 episodes of facial and neck angioedema | NA |
| Ren, 2007 [76] | NA | NA | 18 ± 7 | 16 | ≤1 time/month: 100 (75.2%) 2–3 times/month: 31 (23.3%) >3 times/month: 2 (1.5%) | NA |
| Zhi, 2007 [77] | NA | NA | R: 2–49  Mean: 21.5 | NA | NA | NA |
| Tang, 2009 [36] | NA | 18 | 2 | NA | 8 T/year | NA |
| Lei, 2011 [13] | NA | NA | R: 5–30  20.82 ± 7.88 | 1–39 (8.45 ± 11.04) | NA | NA |
| Xu, 2012 [16] | Group A： patients with disease-causative mutations  Group B： patients with potential deleterious mutations | NA | NA | NA | NA | Clinical severity score range Group A= R: 1–5 Group B= R: 2–6 |
| Qu, 2012 [39] | NA | NA | Proband age = 15 | NA | Monthly angioedema (proband) | NA |
| Xu, 2013 [35] | NA | NA | R: 2–63  Mean = 21.25 | R: 0.1–45  Mean = 12.64 | NA | NA |
| Tang, 2013 [79] | NA | R: 0.5–31  Mean = 15 ± 8 | NA | NA | 19 case records of attack frequency were available as follows:   - 1 case had sporadic episodes - 11 cases had 1–8 episodes/year - 4 cases had 1–2 episodes/month - 3 cases had 1–2 episodes/week | NA |
| Huang, 2013 [80] | NA | 30 | NA | NA | 6~7 times per year | NA |
| Xu, 2014 [21] | NA | NA | Age at onset of upper airway edema  R: 8.0–54.0  27.3 ± 8.7 | NA | NA | NA |
| Zhang, 2014 [82] | NA | 30 | 28 | NA | 10 years ago: 3~4 times/year  in recent 10 years：6~7 times/year  in recent 2 years: >10 times/year | NA |
| Hu, 2014 [83] | NA | 9 | 23 | NA | NA | NA |
| Liu, 2017 [37] | NA | 11 | 15 | NA | NA | NA |
| Li, 2017 [85] | NA | Case 1: > 15  Case 2: > 30 | NA | NA | Case 1: 1–2 episodes per year at the early stage, and later the episodes became frequent  Case 2: 2~3 times/year | NA |
| Liu, 2019 [14] | NA | NA | R: 0–10 y = 15 R: 11–20 y = 52 R: 21–40 y = 29 | C1INH HAE type 1:11.38 (6.33–19.40)  C1INH HAE type 2: 4.88 (2.04–14.65)  • Before 1999: 19.75 (IQR, 13.58–29.50)  • 2000–2009: 8.67 (IQR, 5.67–11.04)  • 2010–2017: 3.79 (IQR, 2.29–5.71) | Median IQR: 8 (3.1–12)/ year | Mild = 11 (11.5%)  Moderate = 11(11.5%) Severe = 74 (77.0%) |
| Liu, 2019 [28] | NA | NA | • 0–10 y: 16 (15.4%) • 11–20 y: 54 (51.9%) • ≥ 21 y: 34 (32.7%) | NA | Median IQR 3.0 (0.8–6.0)/ year | Mild = 39 (37.5%)  Moderate = 20 (19.2%)  Severe = 45(43.3%) |
| Wong, 2019 [40] | NA | NA | Since adolescence | NA | Every 2–3 months | NA |
| Qu, 2019 [87] | NA | 32 | 19 | NA | Once a month | NA |
| Liu, 2020 [20] | NA | NA | R: 0–10 y = 19  R: 11–20 y = 54  R: ≥ 21 y = 34 | NA | NA | Mild = 41  Moderate = 20  Severe = 46 |
| Cao, 2020 [27] | NA | NA | R: 1–40  Mean = 17.5 | Median IQR= 11 (6–19.5) | Median (IQR) Skin: 6 (3–14)/year GI tract: 2 (0–5)/ ear Laryngeal: 0.5 (0–2)/ear | Median IQR severity score = 23 (9.5–45) |
| Cao, 2021 [15] | NA | NA | NA | R: 0–50  Mean = 14.2 | Mean (range) Skin: 9.2/year (0.75–25)  GI tract: 4.6/year (0.12–26) Laryngeal: 2.1/year (0.1-14) | Severity score median IQR  Skin: 8 (5–9) G. I tract: 8.25 (6–9) Laryngeal: 8.75 (6.75–9) |
| Zhang, 2021 [32] | Discovery cohort; validation cohort | NA | NA | NA | NA | Discovery cohort: untreated HAE: clinical severity score R: 2–5 Treated HAE: clinical severity score range R: 2-5   Validation cohort: untreated HAE: clinical severity score R: 1–6 Treated HAE clinical severity score range R: 0-6 |
| Lin, 2021 [38] | NA | 12 | 19 | NA | Once every 3–4 months | NA |
| Wong, 2022 [19] | Total = 44 HAE-index = 15 HAE-screened = 29  Total symptomatic HAE = 30 Symptomatic HAE-index = 15 Symptomatic HAE-screened = 15 | NA | Median = 21.5 (15.0–34.3) Median = 18.0 (15.0–26.0) Median = 30.0 (20.0–35.0) | Median = 10 (0–25.8) Median = 21 (12.0–31.0) Median = 0 (0–23.0)  Median = 21.5 (7.8–31.0) Median = 21.0 (12.0–31.0) Median = 22.0 (27.0–31.0) | NA | NA |
| Wang, 2022 [30] | NA | NA | 18.4 ± 9.4 | NA | NA | NA |
| Cui, 2022 [33] | NA | Patient 1: 31  Patient 2: 18 | Patient 1:20  Patient 2: 17 | NA | Patient 1: < 10 times/year Patient 2: NA | NA |
| Yao, 2022 [88] | NA | 7 | 40 | NA | First onset to 1 year before medication: 6–7 times/year 1 year before medication: 1 time/month | NA |
| Wang, 2022 [45] | NA | NA | NA | NA | Median IQR 5 (1–14)/year | severity score using VAS scale = 7.3 ± 1.5 |
| Jia, 2022 [89] | NA | 12（proband） | 36 | NA | Once every 2 months | NA |
| Wong, 2023 [18] | Garadacimab = 8  Lanadelumab = 3 | NA | Total =18.5 ± 4.2 Garadacimab = 19.3 ± 4.6 Lanadelumab = 16.7 ± 2.9 | NA | Baseline before treatment: 2.5 ± 1.3/ month  After 6 months of treatment: 0.1 ± 0.1/ month | NA |
| Li, 2023 [24] | NA | 42 | 15 | 42 | Once every 1–2 weeks in the last 2 years | NA |
| Lu, 2024 [25] | NA | 20 | 28 | NA | Every month in the past 3 years | NA |
| Yao, 2024 [41] | NA | NA | Patient 1: 18  Patient 2: 10  Patient 3: 16  Patient 4: 20  Patient 5: 16  Patient 6: 12 | Patient 1: 12  Patient 2: 30  Patient 3: 39  Patient 4: 8  Patient 5: 12  Patient 6: 13 | Pre-lanadelumab treatment:  Patient 1: 4 attacks/Year  Patient 2: 14 attacks/Year  Patient 3: 18 attacks/Year  Patient 4: 15 attacks/Year  Patient 5: 25 attacks/Year  Patient 6: 15 attacks/Year  Post-lanadelumab treatment  Patient 1: 0 attacks/Year  Patient 2: 0 attacks/Year  Patient 3: 1 attacks/Year  Patient 4: 0 attacks/Year  Patient 5: 0 attacks/Year  Patient 6: 1 attacks/Year | NA |
| Wang, 2024 [92] | NA | NA | R: 19-34  26.4 ± 5.3 | 15.82 ± 12.36 (1.17–40.08) | GI symptom in the year before diagnosis: 2.13 ± 1.54 (0–4) times  Any attack in the past year: 2.75 ± 1.71 (0–5) times | NA |
| Wang, 2024 [93] | NA | > 3 | 33 | NA | NA | NA |
| Gao, 2024 [94] | NA | NA | 24.00 ± 12.59 | 11.72 ± 12.49 | NA | NA |
| Zhang, 2024 [95] | NA | 11.45 ± 10.36 | NA | NA | 8.64 ± 5.32 (within 1 year) | NA |
| Yao, 2024 [96] | NA | 20.2 | NA | NA | NA | NA |
| Zhou, 2024 [53] | NA | > 7 | 32 | NA | GI symptom: 1~2 times per year | NA |
| Yu, 2024 [58] | NA | NA | NA | R: 5–40  Mean=20 | NA | NA |
| Jiang, 2024 [59] | NA | Case 1: 9  Case 2: > 5 | Case 1 = 8  Case 2 = 26 | NA | Case 1  Before lanadelumab treatment: intervals between edema episodes vary.  After 5 months of treatment: free of attacks  Case 2  Before diagnosis: intervals between edema episodes vary.  Refuse LT treatment: 2 attacks occurred within 7 months after diagnosis | NA |
| Zhang, 2024 [49] | NA | 9 | 8 | NA | 2-3 times/month | NA |
| Xia, 2024 [52] | NA | Case 1: 8  Case 2: >2 | Case 1: 4  Case 2: 15 | NA | Case 1: irregular frequency  Case 2: once every 3~4 months | NA |

*F*, Female; *HAE*, hereditary angioedema; *M*, male; *NA*, not available; *R*, range; *VAS*, visual analogue scale

**Appendix Table 3 Poor Prognosis reported across the studies**

| **Author name, year** | **Year** | **Number of cases (probands)** | **Total number of cases (probands and families)** | **Poor prognosis (proband and lineage)** | **Deaths** | **Age at death** |
| --- | --- | --- | --- | --- | --- | --- |
| Xie, 1980 [50] | 1980 | 1 | 3 | One patient died from HAE | 1 | 69 |
| Zhou, 1980 [100] | 1980 | 1 | 5 | Two patients experienced acute asphyxiation  (patient's mother and sister) | 2 | NA |
| Huang, 1983 [101] | 1983 | 1 | 4 | The patient died of suffocation caused by acute laryngeal edema | 1 | 28 |
| Zhang, 1985 [102] | 1985 | 6 | 25 | Three patients died;  Two died from acute laryngeal edema, and the other's cause of death was unknown | 2 | NA |
| Qian, 1986 [99] | 1986 | 3 | 17 | The patient's grandfather, father, uncle, and sister all died of laryngeal edema | 4 | NA |
| Zhang, 1988 [98] | 1988 | 1 | 14 | Two patients died of laryngeal edema | 2 | NA |
| Qian, 1988 [63] | 1988 | NA | Six hospitalized patients came from three families, and the investigation found a total of 18 patients | Four died from laryngeal edema | 4 | NA |
| Lin, 1989 [62] | 1989 | 1 | 2 | Severe laryngeal edema leading to suffocation and breathing problems | 1 | 59 |
| Zhang, 1990 [64] | 1990 | 24 | 104 | 14 patients died of laryngeal edema | 14 | NA |
| Qian, 1990 [65] | 1990 | 1 (case 2) | 11 | Four patients died of laryngeal edema | 4 | NA |
| Pu, 1991 [66] | 1991 | 2 | 9 | Case 2: The patient's father died of asphyxiation caused by acute laryngeal edema after a tooth extraction | 1 | NA |
| Xie, 1994 [68] | 1994 | 1 | 3 | The patient's father died of suffocation due to breathing difficulties during the fourth attack at the age of 69 years | 1 | 69 |
| Wang 1995 [70] | 1995 | 1 | 5 | The proband's maternal grandfather and mother died young due to laryngeal obstruction | 2 | NA |
| Wang, 1995 [69] | 1995 | 12 | 53 | 10 patients died of HAE | 10 | NA |
| Zhou, 1996 [71] | 1996 | 1 | 10 | The patient’s grandfather, uncle, and sister died of suffocation at the ages of 60, 22, and 34 years, respectively | 3 | 60, 22, and 34 |
| Choy, 1997 [22] | 1997 | 2 | 8 | Two patients died at the ages of 36 and 40 years after an upper airway obstruction during an acute attack of angioedema | 2 | 36, 40 |
| Lu, 2001 [72] | 2001 | 1 | 4 | The patient's grandfather, father, and uncle died of HAE | 3 | NA |
| Zhi, 2003 [48] | 2003 | 1 | 7 | The patient's father died of asphyxiation caused by laryngeal edema | 1 | NA |
| Huang, 2005 [23] | 2005 | 1 | 8 | The patient’s uncle died of a sudden onset of dyspnea at the age of 30 years | 1 | 30 |
| Ashrafian, 2005 [47] | 2005 | 5 | 5 | One patient died at the age of 26 years from acute laryngeal edema | 1 | 26 |
| Sheng, 2005 [74] | 2005 | 2 | 5 | Patient 1’s father (also a close relative) died of suffocation caused by delayed treatment | 1 | NA |
| Peng, 2006 [75] | 2006 | 1 | 6 | The patient's eldest sister died of suffocation at the age of 40 years | 1 | 40 |
| Wen, 2007 [12] | 2007 | 1 | 7 | The patient’s uncle had recurrent laryngeal edema and facial angioedema and died of asphyxia | 1 | NA |
| Lei, 2011 [13] | 2011 | 19 | 19 | Five patients experienced facial or laryngeal attacks. Two patients survived life-threatening attacks after tracheostomy, but one later died from a subsequent attack | 1 | NA |
| Huang, 2013 [80] | 2013 | 1 | 6 | Six individuals in three generations had similar symptoms; the patient's son died of sudden breathing difficulties and suffocation | 1 | 12 |
| Xu, 2013 [35] | 2013 | 158 | NA | 18 patients died from asphyxia caused by laryngeal edema | 18 | NA |
| Zhang, 2014 [82] | 2014 | 1 | 15 | Patients II3 and II9 died of suffocation at the ages of 45 and 34 years, respectively. | 2 | 45, 34 |
| Xu, 2014 [21] | 2014 | 43 | NA | Five patients died of asphyxiation secondary to laryngeal edema | 5 | NA |
| Hu, 2014 [83] | 2014 | 1 | 8 | The patient's maternal grandfather and aunts died of sudden laryngeal edema and suffocation at the ages of 53 and 44 years, respectively | 2 | 53, 44 |
| Wu, 2015 [84] | 2015 | 1 | 3 | The patient's father died of laryngeal edema and suffocation (60 years of age)  The patient's eldest daughter developed dyspnea and laryngeal edema and died of suffocation (30 years of age) | 2 | 60, 30 |
| Li, 2017 [85] | 2017 | 1 (case 1) | 6 | Patient II1 had laryngeal edema at the age of 19 years and died because of delayed rescue | 1 | 19 |
| You, 2017 [86] | 2017 | 1 | 4 | The patient's mother and aunt both died of suffocation caused by delayed rescue | 2 | NA |
| Liu, 2017 [37] | 2017 | 1 | 3 | The proband’s mother died of laryngeal edema | 1 | NA |
| Qu, 2019 [87] | 2019 | 1 | 10 | One patient died of asphyxiation caused by laryngeal edema | 1 | NA |
| Cao, 2020 [27] | 2020 | 103 | 223 | 30 patients died of laryngeal edema | 30 | 46 (IQR 35–53) |
| Jia, 2022 [89] | 2022 | 1 | 9 | One patient died of laryngeal obstruction | 1 | NA |
| Cui, 2022 [33] | 2022 | 2 | NA | Patient 2 died because of systemic organ failure following hypoxic ischemic encephalopathy and pulmonary infection | 1 | 35 |
| Zhang, 2023 [91] | 2023 | 1 | 9 | The patient's uncle died of laryngeal edema and dyspnea after a poor response to hormone shock therapy | 1 | NA |
| Xia, 2024 [52] | 2024 | 2 | 10 | The father of proband 1 died of “sudden asphyxiation” | 1 | NA |
| Liu, 2024 [42] | 2024 | 1 | 3 | The patient had laryngeal edema and died because of complications, including cerebral edema, pulmonary infection, cerebral infarction, and central respiratory and circulatory failure | 1 | 22 |
| **Total deaths** | | | | | 134 | |

*HAE*, hereditary angioedema; *IQR*, inter-quartile range; *NA*, not available

**Appendix Table 4 Complement profile levels in included studies**

| **Author Name, year** | **C1/C3 /C1q levels** | **C4 levels** | **C1INH protein levels** | **C1INH function** |
| --- | --- | --- | --- | --- |
| Zhang, 1988 [98] | C3 normal range: 49–  111 U/mL  Case 1:NA  Case 2: 75 U/mL | Normal range: NA  Case 1: 11.2 mg/dL  Case 2: 14 mg/dL | Normal range: NA  Case 1: 4.6 mg/dL  Case 2: 4.6 mg/dL | Normal range: NA  NA |
| Wang, 1995 [70] | Normal range: NA  NA | Normal range: 0.1–  0.4 g/L  0.09 g/L | Normal range: NA  NA | Normal range: NA  NA |
| Huang, 2005 [23] | Normal range: NA  C3: 119 mg/dL 94 mg/dL 142 mg/dL 145 mg/dL  148 mg/dL 102 mg/dL 100 mg/dL 116 mg/dL 115 mg/dL 134 mg/dL 129 mg/dL | Normal range: NA  12.9 mg/dL 6 mg/dL 47.2 mg/dL 5.4 mg/dL 12.4 mg/dL 11.2 mg/dL 25.9 mg/dL 38.6 mg/dL 42.5 mg/dL 5.4 mg/dL 13 mg/dL | Normal range: NA  59.3 mg/L 48.4 mg/L 205 mg/L 38 mg/L  59.3 mg/L 53.8 mg/L 238 mg/L 247 mg/L 164 mg/L 88.8 mg/L 95 mg/L | Normal range: NA  • Functionally inactive • Functionally inactive • Normal activity • Functionally inactive • Functionally inactive • Functionally inactive • Normal activity • Normal activity • Normal activity • Functionally inactive • Functionally inactive |
| Ren, 2005 [73] | Normal range: NA  NA | Normal range (rate nephelometry assay): 0.10–  0.40 g/L  Normal range (single immunodiffusion assay): 6.470 ±1.880 g/L  HAE (rate nephelometry assay) = 0.163 (0.114) g/L HC (rate nephelometry assay) = 0.270 (0.132) g/L HAE (single immunodiffusion assay) = 3.589 ± 1.932 g/L HC (single immunodiffusion assay) = 5.815 ± 1.934 g/L | Normal range (rate nephelometry assay): 0.21–  0.39 g/L  Normal range (single immunodiffusion assay): 2.333 ±0.390 g/L  HAE (rate nephelometry assay) = 0.064 (0.083) g/L HC (rate nephelometry assay) = 0.229 ± 0.083 g/L HAE (single immunodiffusion assay) = 1.126 ± 0.358 g/L HC (single immunodiffusion assay) = 2.763 ± 0.922 g/L | Normal range: NA  NA |
| Ren, 2007 [76] | Normal range: NA  NA | Normal range: NA  NA | Normal range: NA  Low =130 cases (HAE-1) slightly higher than normal= 3 patients (HAE-2) | Normal range: NA  Low =130 cases (HAE-1)  2% of the normal value= 3 patients (HAE-2) |
| Zhi, 2007 [77] | Normal range: NA  NA | Normal range: 647±188 mg/L  223 ± 89 mg/L | Normal range: 233 ± 39 mg/L  61 ± 23 mg/L | Normal range: NA  NA |
| Ren, 2007 [61] | Normal range: NA  NA | Normal range: 0.10–0.40 g/L  HAE remission period=0.08 ± 0.05 g/L  HAE acute attack period= 0.06 (0.08) g/L  HC= 0.21 ± 0.09 g/L | Normal range: 0.18–0.32 g/L  (median IQR)  HAE remission period= 0.10 (0.06) g/L  HAE acute attack period= 0.06 (0.08) g/L  HC= 0.24 ± 0.03 g/L | Normal range: 0.70–1.30 U/mL  HAE remission period= 0.10 ± 0.07 U/mL HAE acute attack period= 0.08 ± 0.05 U/mL HC= 1.13 ± 0.05 U/mL |
| Tang, 2009 [36] | NA | Normal range: 0.1–0.4 g/L  0.06 g/L | Normal range: 0.21–0.39 g/L  0.056 g/L | Normal range: 0.7–1.3 U/mL  0.12 U/mL |
| Lei, 2011 [13] | Normal range: NA  C3: normal in all patients | Normal range: NA  Low in all patients | Normal range: NA  <50% normal in all patients | Normal range: NA  <50 % of normal (n=7) |
| Xu, 2012 [16] | NA | Normal range: NA  Group A range: (0–0.4583) % Group B range: (0.1177-0.7792) % | Normal range: NA  Group A range: (0.0–0.33) % Group B range: (0.1–0.7725) % | Normal range: NA  Group A range: (0–0.37) U/mL Group B range: (0.1–0.46) U/mL |
| Qu, 2012 [39] | NA | Normal range: (0.16–0.47 g/L)  3^rd^ generation,  1^st^ patient = 0.12 g/L 3^rd^ patient = 0.10 g/L 4^th^ generation,  3^rd^ patient = 0.04 g/L 7^th^ patient = 0.13 g/L | Normal range: (0.21–0.39 g/L)  3^rd^ Generation,  1^st^ patient = 0.0980 g/L 3^rd^ patient = 0.0711 g/L  4^th^ generation,  3^rd^ patient =0.1080 g/L 7^th^ patient = 0.0645 g/L | Normal range: (0.70–1.30 U/mL)  3^rd^ generation,  1^st^ patient = 0.07 U/mL 3^rd^ patient = 0.19 U/mL 4th generation,  3^rd^ patient = 0 U/mL 7^th^ patient = 0.10 U/mL |
| Xu, 2013 [35] | Normal range: NA  Normal anti-C1q level (all patients) | NA | NA | NA |
| Tang, 2013 [79] | Normal range: NA  NA | Normal range: 45.9–83.5 mg/L before 2004, 0.1–0.4 g/L after 2004  17 cases before 2004:  R: 2.2–52.5 mg/L, mean = 23.5 ±12.6 mg/L five cases after 2004:  R:0.05–0.07 g/L, mean = 0.06 ± 0.01 g/L | Normal range: 0.21–0.39 g/L  R: 0.06–0.152 g/L  Mean = 0.08 ± 0.06 g/L | Normal range: 0.7–1.3 U/mL  R: 0.1–0.18 U/mL  Mean = 0.14 ± 0.04 U/mL |
| Liu, 2017 [37] | Normal range: NA  C3: 110 mg/dL | Normal range: NA  11 mg/dL | Normal range: 15–35 mg/dL  8 mg/dL | NA |
| Wong, 2019 [40] | Normal range: NA  : normal | Normal range: NA  C4 level low | Normal range: 0.224–0.387 mg/mL  <0.03 mg/mL | Normal range: 0.7–1.3 U/mL  0.12 U/mL |
| Xu, 2020 [34] | Normal range: NA  NA | Normal range: 0.1–0.4 g/L  NA | Normal range: 0.21 – 0.39 g/L  proband 1 = 0.03 g/L  proband 2 = 0.09 g/L  proband 3 = 0.04 g/L  proband 4 = 0.05 g/L,  proband 5 = 0.04 g/L  proband 6 = 0.04 g/L,  proband 7 = 0.04 g/L  proband 8 = 0.09 g/L,  proband 9 = 0.08 g/L | Normal range: NA  NA |
| Zhang, 2021 [32] | NA | Normal range: 0.100–0.400g/L  **Median (IQR)** Discovery cohort: Untreated HAE = 0.078 (0.056–0.129) g/L Treated HAE = 0.098 (0.062–0.159) g/L Validation cohort: Untreated HAE = 0.032 (0.014–0.066) g/L Treated HAE = 0.062 (0.052–0.095) g/L | Normal range: 0.21–0.39g/L  **Median (IQR)** Discovery cohort: Untreated HAE = 0.080 (0.050–0.110) g/L Treated HAE = 0.075 (0.058–0.123) g/L Validation cohort: Untreated HAE = 0.050 (0.040–0.060) g/L Treated HAE = 0.060 (0.055–0.095) g/L | NA |
| Lin, 2021 [38] | Normal range: NA  C3: 115 mg/dL | Normal range: NA  2 mg/dL | Normal range: 0.21–0.39 g/L  0.054 g/L | NA |
| Huang, 2021 [11] | NA | NA | Normal range: NA  reduced levels | Normal range: NA  reduced function |
| Xu, 2022 [29] | NA | Normal range: NA  Low at baseline | Normal range: NA  Increased from 26% at baseline (normal) to 42% after 6 months of danazol administration, slightly decreasing to 35% at the 5^th^ year, following a rise again to 50% after >10 years of treatment (**%=percentage of the mean of the normal reference value**) | NA |
| Cui, 2022 [33] | NA | Normal range: 0.16 g/L–0.38 g/L  C4 = 0.05 g/L (patient 1)  C4 = 0.05 g/L (patient 2) | Normal range: 0.21–0.39 g/L  Patient 1= 0.04 g/L | Normal range: 0.7–1.3 IU/mL  Patient 2 = 0.01 IU/mL |
| Yao, 2022 [88] | Normal range: NA  NA | Normal range: 100–400 mg/L  <20 mg/L at diagnosis  42 mg/L at the visit | Normal range: 0.21-0.39 g/L  0.05 g/L | Normal range: NA  NA |
| Li, 2023 [24] | NA | Normal range: 0.145–0.360g/L  0.073 g/L | Normal range: 0.21–0.39g/L  0.06 g/L | NA |
| Luo, 2023 [43] | Normal range for C3: 0.80–1.60 g/L C3 = 1.56 g/L  Normal range for C1q: 0–10 U/mL; C1q = 2 U/mL | Normal range: 0.10–0.40 g/L  0.03 g/L | Normal range: 0.21–0.39 g/L  0.09 g/L | Normal range: 68–100%  39.90% |
| Wong, 2023 [44] | NA | Normal range: NA  Low C4 levels | Normal range: 19–37 mg/dL  6 mg/dL | NA |
| Lu, 2024 [25] | NA | Normal range: 14–36 mg/dL  4.5 mg/dL (low) | Normal range: 8–29 mg/dL  0.625 mg/dL | Normal range: >58.9%  7% |
| Lu, 2024 [26] | NA | NA | NA | Normal range: 58.9%  <7% |
| Jindal, 2024 [31] | NA | NA | Normal range: 21-39 mg/dL  52.2 ± 20.0 mg/dL | NA |
| Yao, 2024 [41] | NA | Normal range: 0.1 ~ 0.4g/L  Patient 1 = 54mg/L Patient 2 = <20mg/L Patient 3 = 43mg/L Patient 4 = 60mg/L Patient 5 = 76mg/L Patient 6 = 90mg/L | Normal range: 0.21 ~ 0.39g/L  Patient 1 = 0.08g/L Patient 2 = <0.02g/L Patient 3 = 0.06g/L Patient 4 = 0.07g/L Patient 5 = 0.37g/L Patient 6 = 0.10g/L | Normal range: NA  Patient 1 = 63.3% Patient 2 = 47.4% Patient 3 = 0.0% Patient 4 = 0.2% Patient 5 = 6.0% Patient 6 = 12.2% |
| Wang, 2024 [92] | Normal range: NA  C1q: Normal range | Normal range: NA  Attack period: 0.034 ± 0.017 g/L | Normal range: NA  0.051 ± 0.017 g/L | Normal range: NA  2.8% (0, 17.7%) |
| Wang, 2024 [93] | C3 normal range: 0.7–1.4 g/L  1.22 g/L | Normal range: 72.85–372.95 μg/mL  15.14 μg/mL | Normal range: 81.46–291.29 μg/mL  15.12μg/mL | Normal range: ≥58.9%  <7.0% |
| Zhou, 2024 [53] | Normal range: NA  NA | Normal range: 0.100 ≥ 0.400 g/L  0.068 g/L | Normal range: 0.21–0.39 g/L  0.05 g/L | Normal range: NA  NA |
| Jiang, 2024 [59] | Normal range: NA  NA | Non-attack period normal range: 0.1≥0.4 g/L  Case 1 = 0.06g/L Case 2 = 0.10g/L | Normal range: 0.21–0.39g/L  Case 1 = 0.35 g/L Case 2 = 0.07 g/L | Normal range: ≥68% Case 1= 0%  Case 2= 0% |
| Zhang, 2024 [49] | Normal range: NA  NA | Normal range: 72.85–372.95 ug/ml  19.37 μg/mL | Normal range: 81.46–291.29 ug/ml  25.78 μg/mL | Normal range: ≥58.9  ＜7% |
| Liu, 2024 [42] | NA | Normal range: 0.1–0.4 g/L  0.075 g/L | NA | NA |
| Xia, 2024 [52] | Normal range: NA  NA | Normal range: 0.13–0.37 g/L  Case 1: 0.06 g/L  Case 2: 0.09 g/L | Normal range: 0.21–0.39 g/L  Case 1 = 0.46 g/L Case 2 = 0.05 g/L | Normal range: NA  Case 1 <0.01 Case 2 <0.01 |

*C*, complement; C1INH, C1 esterase inhibitor; *HC*, healthy control; *IQR*, interquartile range; *NA*, not available

**Appendix Table 5 Results of Bradykinin challenge test, D-dimer test, and common misdiagnosis**

| **Author name, year** | **Bradykinin challenge test/D-dimer test** | **Common misdiagnoses** | **chronic spontaneous urticaria**  **no, %** |
| --- | --- | --- | --- |
| Liu, 2019 [14] | NA | Acute gastroenteritis, allergies (unspecified), urticaria, gastrointestinal disease (unspecified), and appendicitis | NA |
| Cao, 2021 [15] | NA | Gastroenteritis, appendicitis, pancreatitis, kidney problems, peritonitis, and gastric perforation  Misdiagnosis of abdominal symptoms led to unnecessary appendectomy or other laparotomy in 19 patients | NA |
| Mak, 2024 [17] | NA | NA | Angioedema and hives 95 (100.0%) Angioedema only 0 (0.0%) |
| Wang, 2024 [92] | D-dimer: 8.2 ± 5.1 mg/L | Acute gastroenteritis and appendicitis | NA |
| Zhou, 2024 [53] | D-dimer: 1.19 mg/L FEU | Eosinophilic gastroenteritis, intestinal obstruction, peritoneal effusion, mesenteritis, peritonitis, and colitis | NA |
| Yu, 2024 [58] | NA | Allergic angioedema, appendicitis, infection, and peptic ulcer | NA |
| Zhang, 2024 [49] | NA | Acute allergies and acute gastroenteritis | NA |

*NA*, not available

**Appendix Table 6 Gene mutations in HAE cases reported across included studies**

| **Author name, year** | **SERPING1 gene mutations** | ***C1INH* gene mutations** | **Other mutations** |
| --- | --- | --- | --- |
| Lei, 2011 [13] | NA | mutation c.628delA, I210fsX210 in exon 5: 6 patients mutation c.3_73del, p. N1fsX34 in exon 3: 3 patients | NA |
| Qu, 2012 [39] | NA | frame-shift mutation c.1391-1445del55 in the exon 8 of the *C1INH* gene from the proband and other affected individuals | NA |
| Xu, 2012 [16] | NA | *35 different mutations comprising 6 nonsense, 10 frameshift, 2 splicing defects, 15 missense, and 2 in-frame mutations.  *25 of these mutations was novel  * 7 SNPs identified, 3 were described for the first time  *Frequencies of each type of mutation were listed in the following order: missense and in-frame (51.21%), frameshift (31.7%), nonsense (14.63%), and splicing defect (4.87%). Exon 8 (36.58%) was the most frequent site of mutations, followed by exon 3 (24.39%), exon 5 and 7 (14.63%,14.63%), exon 6 (4.87%), exon 4 (2.43%), and intron 4 and 6 (2.43%, 2.43%) | NA |
| Liu, 2017 [37] | NA | *C1INH* gene exon 4 c.628+ 1G>T mutation | NA |
| Wong, 2019 [40] | NG 009625. 1: g.12156 14164del2009 mutation | NA | NA |
| Xu, 2020 [34] | 9 mutations were identified  6 of them were novel |  | NA |
| Lin, 2021 [38] | NA | *C1INH* gene exon 4 c.660dupA mutation | NA |
| Jia, 2022 [89] | c.890–2A>G.  (All family members with a history of edema carried this mutation) | NA | NA |
| Wang, 2022 [30] | *35 variants were novel  Missense and in-frame variants were the most common variants (36.8%), followed by frameshift (28.9%), nonsense (14.5%), splice site (13.2%) variants, and gross deletions/duplications (6.6%) | NA | NA |
| Peng, 2023 [90] | heterozygous (c.905del) | NA | NA |
| Li, 2023 [24] | heterozygous missense mutation in SERPING1 (c.1193T > C p. Leu398Pro) | NA | NA |
| Lu, 2024 [26] | mutation (CGC/CAC) at codon 1397 of exon 8 in the SERPING1 gene. | NA | NA |
| Yao, 2024 [41] | **Patient 1**: Heterozygous variant in the SERPING1 gene (c.673_675del [p.F225del]) **Patient 2**: Heterozygous deletion variation in the SERPING1 gene (Seq [GRCh37] del11)(q12.1q12.1), Heterozygous variant in the APOB gene (c.10579C >T [p.R3527W]) **Patient 3**: Heterozygous mutation in the SERPING1 gene at c.1193T > C [p.Leu398Pro] **Patient 4**: NA **Patient 5**: Heterozygous variant in the SERPING1 gene (c.1396C > T [p.R466C])  **Patient 6**: Heterozygous variant in the SERPING1 gene (c.410C > G [p.S137*]) | NA | NA |
| Xia, 2024 [52] | Case 1: c.1396C>G Case 2: c.882C>G | NA | NA |
| Zhang, 2024 [49] | Frameshift heterozygous mutation (chr11:57365749) | NA | NA |
| Du, 2024 [60] | Frameshift heterozygous mutation: 1 (c.933-934insGGAA) Missense heterozygous mutation: 3 (c.1067T＞A, c.1034G＞A, c.1396C＞T) | NA | NA |
| Xu, 2024 [54] | SERPING1+MYOF and SERPING1+HS3ST6 mutations | NA | *SERPING1+MYOF* and *SERPING1+HS3ST6* mutations |
| Wei, 2024 [55] | NA | NA | PLG gene mutation=1 patient |
| Yu, 2024 [58] | 1 patient had a heterozygous mutation in the SERPING 1 gene | NA | NA |
| Wang, 2024 [92] | 3 patients had mutations in the SERPING1 gene | NA | F12 gene=1 Patient |
| Lu, 2024 [25] | missense mutations (c.52-2A>C) | NA | NA |

*C1INH*, C1 inhibitors; *HS3ST6*, heparan sulfate-glucosamine 3-O-sulfotransferase 6 genes; *MYOF*, myoferlin; *NA*, not available; *SERPING1*, serpin family G member

**Appendix Table 7 Treatment and adverse events**

| **Author name, year** | **Treatment received** | **Switching between treatments** | **Dosage** | **Treatment outcomes** | **Adverse events because of treatment** | **Purpose of treatment** |
| --- | --- | --- | --- | --- | --- | --- |
| Zhou, 1980 [100] | Dexamethasone | NA | NA | No significant effect | NA | On-demand |
| Zhang, 1985 [102] | Antihistamine = 7 | NA | NA | No significant effect | NA | On-demand |
| Qian, 1986 [99] | Hydrocortisone, prednisone, dexamethasone, chlorpheniramine, adrenaline, and antibiotics | NA | NA | NA | NA | On-demand |
| Qian, 1990 [65] | Danazol | NA | **Case 1:** 100 mg/d (increased to 150 mg/d during subcutaneous edema attacks)  **Case 2**: 100–200mg/d | No laryngeal edema experienced  Mild episodes of subcutaneous edema were reported | NA | On-demand and long-term prophylaxis |
| Zhi, 2003 [48] | Antihistamine  Danazol | NA | NA | Antihistamine: ineffective  Danazol: effective | NA | On-demand |
| Huang, 2005 [23] | Antihistamine  Steroid | NA | NA | No improvement | NA | NA |
| Ren , 2007 [76] | Danazol = 58 | NA | <100 mg/day = 25 100-200 mg/day = 33 | Good efficacy in all patients and were well-tolerated by most of them | Increased facial oil secretion, acne, weight gain, menstrual disorders in female patients, a mild increase in transaminase (1), and liver cyst (1) | Long-term prophylaxis |
| Zhi, 2007 [77] | Danazol = 45 | NA | Initial dose was 200 mg/T, 3 times/d, used for 3–4 weeks  Maintenance doses were 600 mg/d | *C1INH and C4 were higher than before and C4 reached the normal value *98.6% of patients were able to achieve complete control of HAE (200 mg/time, 3 times/d) | 1 patient discontinued treatment due to side effects; Changes in liver function (28.9%); Menstrual disorders (87.5%); Weight gain; Increased oil secretion; hirsutism and acne; constipation and abdominal discomfort; severe headache | Long-term prophylaxis |
| Tang, 2009 [36] | FFP  Danazol | NA | 400 mL | Complete resolution of symptoms  within 24 hours | NA | Danazol: long-term prophylaxis; FFP: NA |
| Lei, 2011 [13] | LTP-Danazol = 2 | 1 (F) switched temporarily to tranexamic acid | 200 mg once daily (200 mg tapered to 100 mg) | Symptoms were controlled with danazol and no further acute attacks till end of the study. | The female patient was unable to tolerate the virilizing side effects and shifted prophylaxis temporarily to tranexamic acid | Danazol: long-term prophylaxis |
| Tang, 2012 [78] | FFP (13 patients injected with FFP a total of 16 times) | NA | FFP infusion: 586 ± 337 mL | Symptoms were relieved or improved after FFP infusion | Skin rash = 1 case  Abdominal pain =2 cases | On-demand |
| Tang, 2013 [79] | Danazol | NA | Shock dose = 200 mg 2–3 times/d  After 1 to 4 weeks, the maintenance dose = 30 to 400 mg/d Duration of treatment: 5 (1.1 and 10.3) years | The incidence of hydroderma, abdominal pain, and laryngeal edema dropped The level and function of C1INH in 24 patients increased but did not return to the normal | Occasional abdominal pain after taking the drug: 2 patients  Increased ALT: 2 patients Alopecia: 2 patients Seborrhea: 2 patients Dysthesia: 2 patients Weight gain: 6 patients Menstrual disorders: 6 female patients | On-demand and long-term prophylaxis |
| Hu, 2014 [83] | Danazol | NA | 200 mg (3 times a day)  Gradually reduced to 200 mg every other day within 3 months for maintenance | Danazol: local swelling subsided significantly, no occurrence of swelling during the 6-month follow-up | NA | On-demand and long-term prophylaxis |
| Liu, 2017 [37] | LTP Danazol | NA | 200 mg daily | none reported | none reported | Long-term prophylaxis |
| Liu, 2019 [28] | LTP danazol = 74 | NA | NA | No correlation between HRQoL and danazol dosage | Female patients who took larger dosages of danazol: worse social activity, emotional, and mental status | Long-term prophylaxis |
| Liu, 2020 [20] | Danazol = 89 FFP = 10 Tranexamic acid = 4 | NA | **Danazol**: maintenance dose > 200 mg/day = 34.6%  100-199 mg/d = 38.5%   0-99 mg/day = 26.9%  **FFP:** 400 mL **Tranexamic acid:** 0.5, 1, 1.5, and 3 g/d | **Danazol:** decrease in annual attack frequency and better control of laryngeal and GI involvement  **FFP:** oedema subsided after the FFP; one case experienced oedema aggravation during FFP therapy **Tranexamic acid:** Two patients reported a decrease in their attack frequency, whereas one patient experienced an increase in their attack frequency [skin (1), GI (1), and laryngeal (2) oedema] | **Danazol:** menstrual disorders, weight gain,  osteoarticular pain, headaches, visual deterioration, sebaceous hypersecretion, and abnormal liver function  **FFP:** abdominal pain and pruritus **Tranexamic acid**: menstrual disorders, GI symptoms, blurred vision, and itchy skin | On-demand, short-term, and long-term prophylaxis |
| Cao, 2021 [15] | LTP-Danazol = 67  FFP = 1 | NA | NA | • Frequency of GI edema decreased  • Severity score of abdominal symptoms dropped  • The duration of HAE attacks was shortened | •AE due to danazol n = 50 • Menstrual disorder = 33 • Weight gain = 42 • Arthralgia = 28 | Long-term prophylaxis |
| Lin, 2021 [38] | FFP-short-term control LTP danazol | NA | FFU: 4 U/d Danazol: 200 mg/1 tab twice a day, followed by one tablet daily after 1 month | Poor medicine compliance and swelling reappeared | NA | On-demand and long-term prophylaxis |
| Huang, 2021 [11] | Symptomatic therapy (not specified) | NA | NA | Face and lip swelling improved spontaneously, and no episodes were noted in the next several years | NA | NA |
| Cao, 2022 [46] | Danazol = 51  Tranexamic acid = 2 | NA | NA | *Danazol and tranexamic acid levels reduced the abundance of Proteobacteria and Fusobacteria *Community richness normalized after treatment *Predictive profiling indicated fewer pathogenic bacteria after treatment | NA | Long-term prophylaxis |
| Xu, 2022 [29] | LTP danazol = 74 | NA | **Initial dose**: 600 mg/d (For patients who achieved complete control, it was reduced to 400 mg/d, followed by 200 mg/d)  **Maintenance dosage**  200 mg/d: 41(55%) < 200 mg/d: 16 (22%) 400 mg/d: 13 (18%) 600 mg/d: 4 (5.4%) | Danazol showed satisfactory efficacy | AE = 83 instances of  hepatic impairment, weight gain, seborrhea, acne, and menstrual irregularities | Long-term prophylaxis |
| Wang, 2022 [30] | LTP Danazol  LTP tranexamic acid = 2 | NA | NA | NA | NA | Long-term prophylaxis |
| Yao, 2022 [88] | Icatibant = 1 | NA | 30 mg | Laryngeal swelling completely subsided, and the patient could eat and speak normally  A subsequent laryngoscopy showed that the edema on the surface of the epiglottis had subsided significantly | Mild pain at the injection site, transient erythema, transient dizziness, and forehead sweating | On-demand |
| Wong, 2023 [18] | LTP garadacimab = 8 LTP lanadelumab = 3 | NA | Garadacimab = 400 mg loading dose/followed by 200 mg every 4 weeks Lanadelumab = 300 mg every 2 weeks *No dose adjustments were made | • Frequency of attacks per month reduced after 6 months of treatment • 70% of the patients were attack-free during 6 months • No patients required hospitalization during the 6-month period •Hospital Anxiety and Depression Scale scores remained within the normal range •Significant improvements in work productivity and a reduction in activity impairment  • Significant improvement in treatment satisfaction  • AE-QoL scores improved across all dimensions | NA | Long-term prophylaxis |
| Li, 2023 [24] | On demand: Icatibant LTP lanadelumab | NA | Lanadelumab: 2 injections biweekly | **Icatibant**: Relief from the dyspnea and recovered to normal functioning after 4 days  **Lanadelumab**: none reported | NA | On-demand and long-term prophylaxis |
| Lu, 2024 [26] | Lanadelumab | NA | 300 mg every 4 weeks | Symptom resolution;  rapid normalization of amylase and lipase levels | NA | NA |
| Peng, 2023 [90] | Icatibant = 1 | NA | 30 mg | *Abdominal pain subsided, and abdominal examination was normal.  *No recurrence of abdominal pain during follow-up | NA | On-demand |
| Luo, 2023 [43] | STP: FFP LTP: attenuated androgens Icatibant: for acute treatment of attacks | NA | FFP 2 units LTP: attenuated androgens starting at 400 mg/d and gradually reducing to 200 mg/d  Icatibant: 2 doses | Patients were able to actively engage in normal work and life | NA | On-demand, short-term prophylaxis and long-term prophylaxis |
| Wong, 2023 [44] | LTP: Danazol C1INH replacement therapy: For recurrent attacks | NA | NA | none reported | NA | Long-term prophylaxis |
| Zhang, 2023 [91] | Massive hormone shock and adrenaline therapy FFP | NA | FFP: virus-inactivated plasma 2U | Massive hormone shock and adrenaline therapy: not significantly improved  FFP: all conditions improved, and upon reexamination of the laryngoscope, no significant swelling was observed in the epiglottis proved | NA | On-demand |
| Lu, 2024 [25] | LTP lanadelumab | NA | 300 mg every 2 weeks (6 months), followed by 300 mg every 4 weeks | No recurrence of abdominal pain or edema. | NA | Long-term prophylaxis |
| Yao, 2024 [41] | LTP lanadelumab = 6 | NA | Lanadelumab: 300 mg /once every 2 weeks for 37 weeks | Reduction of 97.8% and 98.5% in the attack rate and treated attack rate, respectively. Significant improvements in the AE-QoL and DLQI scores (100% reduction rates) till the end of the treatment and in missed workdays/year (98.9% reduction rate) | *No serious/severe TEAEs or deaths were observed;   Injection site pain and injection site erythema were the most common TEAE | On-demand  and long-term prophylaxis |
| Wang, 2024 [92] | Lanadelumab = 3 Icatibant = 2 Danazol = 1 | NA | **L:** 300 mg/time, once every 2 weeks. After 6 months, if the condition is fully controlled, it can be changed to 300 mg/time, once every 4 weeks. **I:** 30 mg/time during attack; if the condition is not well controlled, add 30 mg/time after 6 hours **D:** 200 mg/time, oral 2–  3 times/day, gradually reduce the dose to the maintenance dose after 1–4 weeks | **L:** Two patients did not relapse after treatment, and another patient occasionally had skin lesions, but no abdominal pain or laryngeal edema relapsed **I:** One patient experienced relief quickly after taking the medicine, and the other patient did not relapse, so no medicine was used **D**: skin lesions occasionally occurred after taking the medicine, but abdominal pain did not recur | **L**: no drug-related AE **I**: no drug-related AE **D**: obesity, high blood pressure, and increased body hair | I: on-demand  L&D: on-demand and long-term prophylaxis |
| Wang, 2024 [93] | Lanadelumab = 1 | NA | 300 mg once every 2 weeks | The patient's abdominal pain symptoms improved, and there was no recurrence | NA | On-demand and long-term prophylaxis |
| Gao, 2024 [94] | Lanadelumab = 8 | NA | NA | During the regular treatment period, there was no recurrence of symptoms | NA | Long-term prophylaxis |
| Zhang, 2024 [95] | No mention of the drug | NA | NA | After 6 months, the symptom control (AECT) score (14.45 ± 1.13) was higher than that before treatment (5.45 ± 3.53) (*P* < 0.05); the anxiety and depression scores [(6.82 ± 2.60) points, (5.72 ± 2.72) points] were lower than those before treatment [(14.82 ± 1.83) points, (15.09 ± 2.39) points], (*P*< 0.05); the AE-QoL score (24.36 ± 10.65) was lower than that before treatment (56.18 ± 15.94), (*P*< 0.05); the differences were statistically significant | NA | NA |
| Yao, 2024 [96] | Icatibant = 20 | NA | NA | 90% of patients were satisfied with the drug's efficacy and ease of use *All injections were divided into Group A: abdominal attack group; Group B: skin attack group; and  Group C: throat attack group. The average first symptom relief time after injection in groups A, B, and C was shortened to 0.5, 1, and 0.5 hours; the average significant relief time was shortened to 2, 6, and 1.8 hours; the complete relief time was shortened to 4, 16, and 6 hours | NA | On-demand |
| Zhou, 2024 [53] | Danazol = 1 | NA | 0.2 g/once a day | Patients had no recurrence of abdominal pain, diarrhea, or limb swelling. | NA | On-demand and long-term prophylaxis |
| Jiang, 2024 [59] | Lanadelumab = 1 | NA | Lanadelumab injection: 300 mg/time 2 weeks/time  After 3 months, reduced to 3 weeks/time | L: During the treatment period, no edema recurred | No obvious adverse reactions occurred | On-demand and long-term prophylaxis |
| Zhang, 2024 [49] | Lanadelumab = 1 | NA | NA | The patient's condition did not relapse during the nearly two months of the follow-up period | NA | Long-term prophylaxis |

*AE*, adverse events; *C1INH*, C1 inhibitors; *FFP*, fresh frozen plasma; *GI*, gastrointestinal; *LTP*, long term prophylaxis; *NA*, not available; *TEAEs*, treatment-emergent adverse events; *STP*, short term prophylaxis

**Appendix Table 8: QoL questionnaires**

| **Author name, year** | **QoL questionnaire assessed** | **Outcomes** |
| --- | --- | --- |
| Liu, 2019 [28] | SF-36v2 | •SF-36v2 showed a significant reduction in all dimensions of HRQoL (*P* < 0.001) in patients with HAE compared with the general Chinese population •A correlation between decreased disease control and decreased HRQoL scores was observed, although the correlation was not significant in terms of RE or mental health scores |
| Wong, 2022 [19] | AE-QoL and HADS | **AE-QoL: Overall:** Mean difference between T1 and T2: Functioning = –11.5 ± 22.2, Nutrition = 8.3 ± 17.5, Change from baseline = –20.7 ± 18.7 **HAE Index patients**: Mean difference between T1 and T2: Functioning = –21.5 ± 22.8, Nutrition = –13.5 ± 21.3, Change from baseline = –28.7 ± 16.2, Total score = 37.2 ± 26.9,  **HAE SS patients**: Change from baseline = –11.3 ± 17.6, Total score = 31.7 ± 30.8 **HADS:** HADS A time point 1: 12.21 ± 6.36; HADS A after 1 year follow-up: 6.67 ± 4.43; HADS D time point 1: 4.76 ± 4.21; HADS D after 1 year follow-up: 6.00 ± 3.79 HADS A time point 1: 10.92 ± 6.53; HADS A after 1 year follow-up: 6.92 ± 4.94; HADS D time point 1: 6.08 ± 3.59 HADS A time point 1: 13.73 ± 6.10; HADS A after 1 year follow-up: 6.91 ± 4.09; HADS D time point 1: 4.30 ± 3.50; HADS D after 1 year follow up: 5.87 ± 4.87 HADS A time point 1: 15.50 ± 7.15; HADS A after 1 year follow-up: 5.6 ± 4.5; HADS D time point 1: 5.83 ± 6.43; change in HADS D: 0.33 ± 6.77 |
| Wong, 2023 [18] | AE-QoL questionnaire, Hospital Anxiety and Depression Scale, Work Productivity and Activity Impairment Questionnaire: General Health, version 2.0; and Treatment Satisfaction Questionnaire for Medication, version II, were administered just before the initiation of LTP and repeated after 6 months | **AE-QoL:** There were significant improvements across all dimensions of the AE-QoL questionnaire, and an improvement in the overall score exceeded the minimal clinically important difference. LTP also significantly improved the scores on all dimensions of the AE-QoL questionnaire. **The Hospital Anxiety and Depression Scale** scores were within the normal range both at baseline and after LTP **Work Productivity:** Marked improvements in work productivity were observed in several domains of Work Productivity  **Activity Impairment Questionnaire**: General health before treatment, including an 8.5% reduction in work time missed, a 68.6% reduction in impairment while working, a 59.1% reduction in overall work impairment, and a 55.7% reduction in activity impairment  *There was a significant reduction in activity impairment after the use of LTP (from 55.7% to 15.7%). |
| Mak, 2024 [17] | AAS, AE-QoL questionnaire, and AECT | **AAS:** Chinese AAS showed excellent internal consistency (Cronbach’s alpha = 0.920) and was significantly correlated with the AE-QoL QoL (*P* = 0.276) and AECT (P = –0.750). **AE-QoL questionnaire:** Chinese AE-QoL demonstrated excellent internal consistency (Cronbach’s alpha = 0.976), with moderate correlations with generic PROMs, and the Work Productivity and Activity Impairment Questionnaire and SF-12 (*P* < 0.60) showed significant negative correlations with both AAS and AECT scores **AECT:** Chinese AECT showed excellent internal consistency (Cronbach’s alpha = 0.832) and was negatively correlated with both AAS (*P*= –0.750) and AE-QoL (*P* = –0.456), reflecting its role in measuring disease control |
| Yao, 2024 [41] | AE-QoL questionnaire and DLQI scores | **Baseline AE-QoL Scores** (Mean ± SD): 53.9 ± 10.7; Functioning Domain: 45.6 ± 19.2; Fatigue/Mood Domain: 42.5 ± 18.9; Fears/Shame Domain: 75.7 ± 12.8; and Nutrition Domain: 33.3 ± 20.4 *After 4 weeks of lanadelumab treatment, AE-QoL total and domain scores significantly dropped to 0, with the largest improvement in the fears/shame domain. These reductions were sustained through the end of the treatment  **DLQI score:** The mean DLQI score for six patients was 22.3 ± 3.9 at baseline *After 2 weeks of lanadelumab treatment, a profound reduction of 100% was observed, and this reduction was maintained without rebound throughout the subsequent visits |
| Zhang, 2024 [95] | AE-QoL, AECT, anxiety, and depression scores | ***AECT score**: After 6 months of intensive treatment and nursing, the symptom control (AECT) score of 11 patients (14.45±1.13) was higher than that before treatment (5.45 ± 3.53), (*P* < 0.05); ***The anxiety and depression scores** [(6.82 ± 2.60) points, (5.72 ± 2.72) points] were lower than those before treatment [(14.82 ± 1.83) points, (15.09 ± 2.39) points], (*P* < 0.05) ***The AE-QoL score** (24.36 ± 10.65) was lower than that before treatment (56.18 ± 15.94), (P < 0.05); the differences were statistically significant |

*AAS,* Angioedema Activity Score; *AECT*, Angioedema Control Test; *AE-QoL*, Angioedema Quality of Life; *DLQI*, Dermatology Life Quality Index; *HADS*, Hospital Anxiety and Depression Scale; *HRQoL*, health-related quality of life; *NA*, not available; *SF-36v2*, Short Form Health Survey version-2.0; *QoL*, quality of life

**Appendix Table 9 Prevalence of HAE**

| **Author name, year** | **Prevalence rate (%)** | **Comments** |
| --- | --- | --- |
| Lei, 2011 [13] | The prevalence was low in Taiwan | NA |
| Lin, 2021 [38] | 1:550,000–1,000,000 in Taiwan | Reported by another study, which is cited in this study |
| Wong, 2022 [19] | The estimated point prevalence of HAE in Hong Kong increased from 1/ 640,000 to 1/ 166,000 | Reported by another study, which is cited in this study |
| Cui, 2022 [33] | The prevalence of HAE in the C4 decreased cohort was 2.43/10,000 The prevalence of HAE in the C4 decreased cohort (etiology undetermined) was 10.1/10,000 The prevalence of HAE in clinically suspected patients (C4 below normal) was 11.1% | NA |

*NA*, not available
